# Global Trends and Future Projections in the Burden of Inflammatory Bowel Disease Among Adolescents and Young Adults (15–49 Years) From 1990 to 2021

**DOI:** 10.1002/jgh3.70282

**Published:** 2025-09-18

**Authors:** Xueyi Ren, Jun Xu, Xiaolei Zhao

**Affiliations:** ^1^ Department of Gastroenterology Peking University People's Hospital Beijing China; ^2^ Clinical Center of Immune‐Mediated Digestive Diseases, Peking University People's Hospital Beijing China

**Keywords:** age‐standardized rates, global burden of disease, inflammatory bowel disease, prevalence

## Abstract

**Aims:**

Inflammatory bowel disease (IBD) is an immune‐mediated disorder with rising global incidence. Adolescents and young adults (15–49 years) bear major psychological, social, and economic burdens, yet few studies have examined their disease trends. We aimed to estimate global, regional, and national incidence, prevalence, mortality, and disability‐adjusted life years (DALYs) of IBD in this age group and to project future burden.

**Methods and Results:**

Using data from the Global Burden of Disease 2021, we analyzed age‐standardized rates of incidence, prevalence, mortality, and DALYs (ASIR, ASPR, ASMR and ASDR) among people aged 15–49 across 204 countries and territories. Estimated annual percentage changes, Joinpoint regression, and age‐period‐cohort modelling were employed to evaluate temporal patterns, while Bayesian modelling projected trends to 2050. Inequalities were evaluated using the Socio‐demographic Index (SDI). In 2021, global ASIR was 5.01/100,000 and ASPR was 41.56/100,000. ASMR and ASDR were 0.13/10,000 and 13.56/100,000 person‐years, respectively. From 1990 to 2021, ASIR and ASPR increased slightly overall, with the most rapid rise in East Asia. ASMR and ASDR declined globally but remained highest in Western Sub‐Saharan Africa. SDI was positively correlated with incidence and prevalence, and negatively with mortality. Projections to 2050 indicate continued declines in incidence and prevalence, stable DALYs, and a slight increase in mortality.

**Conclusion:**

IBD remains a significant burden in people aged 15–49. Growing incidence in East Asia and sustained mortality in disadvantaged regions highlight the need for early diagnosis, equitable care, and targeted public health strategies.

## 1 Introduction

Inflammatory bowel disease (IBD), which includes Crohn's disease (CD) and ulcerative colitis (UC), is a chronic immune‐mediated condition characterized by persistent inflammation of the gastrointestinal tract [1]. Clinically, IBD presents with a wide range of symptoms such as abdominal pain, diarrhea, hematochezia, fever, and various extraintestinal manifestations [2]. In recent years, the incidence of IBD has continued to rise worldwide. However, due to the complex and multifactorial nature of its pathogenesis, current treatment strategies primarily aim to control symptoms and maintain remission, while a definitive cure remains elusive. As a result, IBD imposes a substantial burden on patients in terms of psychological stress, economic costs, and reduced quality of life.

From an epidemiological perspective, IBD has long been considered a disease primarily affecting Western countries, where higher prevalence has been associated with economic development and industrialization. However, recent studies have shown a shifting trend: while the incidence of IBD has plateaued in many high‐income countries, it is increasing rapidly in newly industrialized regions such as Eastern Europe, Asia, and Africa [3, 4]. These changes may be associated with a variety of factors, including dietary patterns, environmental exposures, and medication use [5].

IBD most commonly affects individuals between the ages of 20 and 40 [6], a demographic that represents the core of the labor force in many societies. Given the chronicity, treatment resistance, and frequent complications associated with IBD [7], the disease can reduce work productivity, increase healthcare expenditures, and contribute to a growing global health burden. Furthermore, IBD may interfere with social and economic development, particularly in countries undergoing demographic and lifestyle transitions [8].

Despite its growing significance, few studies have specifically examined the burden and temporal trends of IBD among adolescents and young adults. Understanding the age‐specific burden of IBD is crucial, as it may inform resource allocation, disease prevention strategies, and long‐term planning. In addition, identifying geographic variations in the incidence, prevalence, and prognosis of IBD may provide valuable insights into its etiology and underlying risk factors.

The most recent estimates from the Global Burden of Disease Study 2021 (GBD 2021), conducted by the Institute for Health Metrics and Evaluation (IHME), provide comprehensive data on the burden of IBD across five Socio‐demographic Index (SDI) regions and 224 countries and territories from 1990 to 2021 [9]. Based on this dataset, the present study aims to assess the disease burden and temporal trends of IBD among individuals aged 15–49 years. Specifically, we analyze age‐standardized incidence rates (ASIR), mortality rates (ASMR), disability‐adjusted life years (DALYs), and estimated annual percentage changes (EAPCs) at the global, regional, and national levels. In addition, we project the future burden of IBD through 2050 to inform long‐term global health strategies.

## 2 Methods

### 2.1 Data Source

This study utilized data from the Global Burden of Disease (GBD) 2021, a comprehensive epidemiological database that systematically estimates the burden of 371 diseases and injuries across 204 countries and territories from 1990 to 2021 [10]. Data can be downloaded from https://vizhub.healthdata.org/gbd‐results/. The study focused on Inflammatory Bowel Disease (IBD), defined by the International Classification of Diseases, Tenth Revision (ICD‐10) codes K50–K51.319, K51.5–K52, and K52.8–K52.9.

IBD‐related data, including prevalence, incidence, mortality, and disability‐adjusted life years (DALYs), were analyzed by age (15–49 years), sex, country, and region. Incidence rate.

(per 100,000) was calculated by dividing the number of new cases by the population size; mortality rate (per 100,000) was defined as the number of annual deaths divided by the total population size; The impact of IBD was quantified using DALYs [11], which represent the number of healthy years lost each year per 100,000 people.

To explore the socioeconomic distribution of IBD burden, the analysis also incorporated the World Bank's income classification and the Socio‐demographic Index (SDI), a composite metric reflecting per capita income, educational attainment, and fertility rates [12, 13]. These indicators allowed for stratified comparisons of IBD burden across development levels. The study's temporal scope spans from 1990 to 2021, capturing long‐term trends in the global epidemiology of IBD among young and middle‐aged adults.

### 2.2 Statistical Analysis

To characterize the global burden of Inflammatory Bowel Disease (IBD) among individuals aged 15–49 years, we use ASR, which stands for the age‐standardized rate, while ASIR refers to the age‐standardized incidence rate, ASPR refers to the age‐standardized prevalence rate, ASMR refers to the age‐standardized mortality rate, and ASDR represents the age‐standardized DALY rate.

These metrics were derived using the Global Burden of Disease (GBD) 2021 estimates standardized to the GBD global reference population. Temporal trends from 1990 to 2021 were evaluated by calculating the Estimated Annual Percentage Change (EAPC) using a log‐linear regression model:

$$y=\alpha +\beta x+\varepsilon ,\;EAPC=100\times \left(e^{\beta }-1\right)$$

and 95% confidence intervals (CIs) were used to determine significance. A positive EAPC and CI indicate an increasing trend, while both negative values indicate a decreasing trend [14].

Further trend analyses employed the Joinpoint regression model developed by the National Cancer Institute [15]. The model identifies significant trend shifts (“joinpoints”) and calculates Annual Percent Change (APC) for each segment, as well as Average Annual Percent Change (AAPC) for the overall period [16]. The Monte Carlo permutation test and Bayesian Information Criterion were used for model selection. When assessing future projections, we applied the Bayesian age‐period‐cohort (BAPC) model using integrated nested Laplace approximations (INLA), assuming multiplicative effects of age, period, and cohort [17, 18].

In addition, to evaluate cross‐national inequalities in IBD burden, we employed the Slope Index of Inequality (SII) and the Concentration Index (CI), which quantify disparities in disease burden in relation to the Socio‐demographic Index (SDI). These indices were calculated through weighted regression and Lorenz curve integration, respectively.

All statistical analyses and visualizations were performed using Microsoft Excel 2021 and R software (version 4.3.3), along with relevant R packages including dplyr, officer, and ggplot2. Data on the global burden of IBD among the adolescents and young adults (15–49 years) from 1990 to 2021 were cleaned and processed prior to analysis. A two‐sided *p*‐value of less than 0.05 was considered statistically significant.

## 3 Results

### 3.1 Global Burden of IBD in the 15–49‐Year‐Old Population

In 2021, the global age‐standardized incidence rate (ASIR) of inflammatory bowel disease (IBD) among individuals aged 15–49 years was 5.01 (95% UI: 3.83–6.52) per 100,000 population, and the age‐standardized prevalence rate (ASPR) was 41.56 (95% UI: 32.62–52.54) per 100,000. The age‐standardized rate (ASR) of disability‐adjusted life years (DALYs) was 13.56 (95% UI: 10.65–16.96) per 100,000 person‐years, and the age‐standardized mortality rate (ASMR) was 0.13 (95% UI: 0.10–0.15) per 10,000. This translates into an estimated 201,894.96 (95% UI: 154,071.05–262,362.69) new IBD cases among people aged 15–49 in 2021, including 102,920.40 (95% UI: 78,417.42–133,660.24) men and 98,974.56 (95% UI: 75,474.80–128,649.18) women. There were approximately 1,684,616.35 (95% UI: 1,322,638.96–2,128,877.99) prevalent cases, comprising 817,618.98 males and 866,997.38 females. In the same year, IBD caused 545,671.42 (95% UI: 428,459.78–682,979.20) DALYs in this age group, with a near‐equal sex distribution (men: 264,091.06; women: 281,580.36). A total of 5,225.83 deaths (95% UI: 4,013.18–6,160.96) were attributed to IBD, including 2,639.07 men and 2,586.76 women. From 1990 to 2021, the EAPC for ASIR remained positive, indicating an overall increasing trend in IBD burden among individuals aged 15–49 years worldwide (Table 1).

**TABLE 1:** Age‐standardized incidence, prevalence, death and DALY rates for inflammatory bowel disease (IBD) in 1990 and 2021 and their temporal trends from 1990 to 2021.

Characteristics	Age‐standardized incidence rate per 100 000 population			Age‐standardized prevalence rate per 100 000 population			Age‐standardized death rate per 100 000 population			Age‐standardized DALY rate per 100 000 population		
	1990 no (95% UI)	2021 no (95% UI)	EAPC no (95% CI)	1990 no (95% UI)	2021 no (95% UI)	EAPC no (95% CI)	1990 no (95% UI)	2021no (95% UI)	EAPC no (95% CI)	1990 no (95% UI)	2021 no (95% UI)	EAPC no (95% CI)
Global	4.88 (3.81, 6.22)	5.01 (3.83, 6.52)	0.21 (0.09, 0.34)	47.13 (38.47, 57.83)	41.56 (32.62, 52.54)	−0.32 (−0.49, −0.14)	0.15 (0.12, 0.18)	0.13 (0.10, 0.15)	−0.65 (−0.72, −0.59)	15.37 (12.28, 19.04)	13.56 (10.65, 16.96)	−0.45 (−0.52, −0.38)
Sex												
Male	4.92 (3.84, 6.26)	5.06 (3.86, 6.58)	0.209 (0.086, 0.332)	44.87 (36.45, 55.07)	39.98 (31.37, 50.56)	−0.282 (−0.457, −0.107)	0.16 (0.13, 0.19)	0.13 (0.10, 0.16)	−0.949 (−1.039, −0.859)	15.27 (12.34, 18.76)	12.97 (10.05, 16.40)	−0.620 (−0.691, −0.549)
Female	4.84 (3.77, 6.19)	4.97 (3.79, 6.46)	0.219 (0.090, 0.348)	49.45 (40.42, 60.81)	43.17 (33.85, 54.55)	−0.350 (−0.519, −0.180)	0.14 (0.09, 0.19)	0.13 (0.09, 0.17)	−0.318 (−0.379, −0.258)	15.46 (11.47, 20.12)	14.16 (10.76, 18.08)	−0.279 (−0.350, −0.209)
SDI quintile												
High SDI	13.89 (11.13, 17.21)	14.50 (11.22, 18.44)	0.16 (−0.02, 0.33)	143.67 (119.49, 173.97)	133.34 (106.01, 167.48)	−0.29 (−0.56, −0.02)	0.13 (0.13, 0.14)	0.12 (0.12, 0.13)	0.04 (−0.18, 0.26)	29.06 (21.15, 38.46)	26.59 (19.10, 35.53)	−0.24 (−0.48, −0.00)
High middle SDI	3.47 (2.68, 4.48)	3.92 (2.96, 5.18)	0.67 (0.44, 0.90)	35.34 (28.48, 43.96)	33.62 (26.39, 42.83)	0.14 (−0.12, 0.40)	0.14 (0.12, 0.16)	0.08 (0.07, 0.09)	−2.38 (−2.63, −2.13)	12.56 (10.38, 15.22)	9.43 (7.32, 12.00)	−1.13 (−1.26, −0.99)
Middle SDI	1.82 (1.35, 2.42)	2.77 (2.07, 3.69)	1.65 (1.43, 1.86)	14.85 (11.53, 19.23)	21.05 (16.31, 27.26)	1.44 (1.24, 1.65)	0.12 (0.09, 0.15)	0.09 (0.07, 0.11)	−1.01 (−1.12, −0.89)	8.89 (6.83, 10.65)	8.35 (6.63, 10.41)	−0.14 (−0.19, −0.08)
Low middle SDI	3.97 (2.98, 5.28)	4.67 (3.50, 6.21)	0.60 (0.50, 0.70)	28.37 (21.84, 36.88)	32.49 (24.82, 42.35)	0.65 (0.54, 0.77)	0.19 (0.13, 0.25)	0.15 (0.11, 0.19)	−0.82 (−0.89, −0.75)	14.79 (11.10, 19.11)	13.60 (10.46, 17.16)	−0.30 (−0.36, −0.24)
Low SDI	2.77 (2.08, 3.70)	3.16 (2.37, 4.20)	0.51 (0.44, 0.57)	20.40 (15.65, 26.39)	21.98 (16.76, 28.80)	0.34 (0.26, 0.41)	0.26 (0.16, 0.36)	0.26 (0.15, 0.35)	−0.17 (−0.25, −0.10)	17.55 (11.77, 23.28)	17.65 (11.41, 22.96)	−0.05 (−0.11, 0.02)
GBD region												
Andean Latin America	1.58 (1.16, 2.14)	1.82 (1.32, 2.46)	0.51 (0.43, 0.58)	14.39 (10.81, 18.96)	14.68 (11.03, 19.29)	0.03 (−0.08, 0.15)	0.08 (0.06, 0.12)	0.05 (0.04, 0.08)	−1.50 (−1.76, −1.23)	7.08 (5.14, 9.65)	5.33 (3.89, 7.23)	0.98 (0.49, 1.48)
Australasia	22.92 (17.49, 30.09)	24.82 (19.12, 32.27)	0.69 (0.36, 1.01)	208.95 (163.79, 270.16)	217.01 (170.77, 277.34)	0.85 (0.34, 1.36)	0.05 (0.04, 0.05)	0.08 (0.07, 0.10)	2.65 (1.74, 3.56)	33.94 (21.18, 51.11)	36.73 (23.60, 53.85)	0.73 (0.44, 1.01)
Caribbean	2.96 (2.20, 3.95)	3.45 (2.57, 4.59)	0.47 (0.40, 0.53)	31.46 (24.63, 40.15)	31.16 (24.07, 40.33)	−0.01 (−0.03, 0.01)	0.26 (0.21, 0.33)	0.19 (0.13, 0.29)	−1.19 (−1.45, −0.93)	18.78 (15.37, 23.03)	15.38 (11.27, 21.09)	0.42 (0.35, 0.50)
Central Asia	5.10 (3.83, 6.74)	6.02 (4.54, 8.02)	0.55 (0.54, 0.57)	47.96 (37.43, 61.34)	49.17 (38.16, 63.72)	0.14 (0.09, 0.18)	0.20 (0.17, 0.22)	0.16 (0.14, 0.20)	−1.23 (−1.52, −0.94)	18.21 (14.87, 22.31)	16.71 (13.15, 21.01)	0.11 (−0.11, 0.34)
Central Europe	7.09 (5.52, 9.13)	8.39 (6.39, 10.86)	0.78 (0.55, 1.01)	78.75 (63.90, 97.46)	81.10 (63.89, 102.04)	0.34 (0.08, 0.61)	0.16 (0.14, 0.17)	0.11 (0.10, 0.12)	−0.94 (−1.13, −0.76)	20.56 (15.91, 26.20)	18.38 (13.58, 24.41)	0.09 (0.02, 0.16)
Central Latin America	0.70 (0.51, 0.94)	0.66 (0.47, 0.90)	0.03 (−0.12, 0.19)	6.09 (4.60, 8.02)	5.84 (4.41, 7.70)	−0.05 (−0.19, 0.08)	0.12 (0.11, 0.12)	0.13 (0.12, 0.15)	0.85 (0.50, 1.19)	7.20 (6.68, 7.83)	7.92 (7.05, 8.88)	0.06 (−0.05, 0.17)
Central sub−Saharan Africa	1.59 (1.19, 2.12)	2.07 (1.56, 2.77)	0.91 (0.89, 0.93)	13.38 (10.26, 17.48)	13.24 (9.99, 17.59)	−0.19 (−0.34, −0.04)	0.15 (0.08, 0.25)	0.15 (0.08, 0.27)	0.16 (0.05, 0.26)	10.40 (6.57, 15.66)	10.51 (6.68, 16.70)	−0.01 (−0.23, 0.21)
East Asia	0.87 (0.64, 1.17)	1.67 (1.24, 2.23)	3.06 (2.40, 3.73)	6.60 (5.00, 8.66)	10.26 (7.82, 13.42)	2.46 (1.70, 3.23)	0.10 (0.06, 0.13)	0.05 (0.03, 0.06)	−2.78 (−2.99, −2.58)	6.21 (4.22, 7.96)	4.05 (3.09, 5.15)	−0.08 (−0.35, 0.19)
Eastern Europe	3.80 (2.84, 5.05)	4.57 (3.40, 6.11)	0.60 (0.57, 0.63)	35.32 (27.50, 45.28)	35.83 (27.51, 46.37)	0.11 (0.01, 0.21)	0.29 (0.26, 0.35)	0.20 (0.18, 0.23)	−2.22 (−2.69, −1.74)	20.15 (17.16, 24.16)	15.91 (13.39, 18.73)	−0.10 (−0.64, 0.44)
Eastern sub−Saharan Africa	1.52 (1.14, 2.02)	1.85 (1.39, 2.45)	0.75 (0.69, 0.81)	11.25 (8.63, 14.52)	12.78 (9.79, 16.62)	0.27 (0.21, 0.33)	0.14 (0.08, 0.20)	0.15 (0.08, 0.21)	0.03 (−0.05, 0.12)	9.31 (6.10, 12.21)	9.83 (6.30, 13.63)	−0.12 (−0.34, 0.10)
High−income Asia Pacific	2.79 (2.13, 3.66)	3.55 (2.68, 4.66)	1.14 (0.51, 1.77)	32.21 (25.46, 40.68)	38.83 (30.53, 49.19)	1.01 (0.35, 1.66)	0.10 (0.07, 0.13)	0.04 (0.03, 0.05)	−3.31 (−3.50, −3.12)	10.60 (8.07, 13.68)	7.99 (5.49, 11.20)	−0.16 (−0.19, −0.12)
High−income North America	21.24 (16.98, 26.35)	22.27 (17.42, 28.04)	0.19 (0.03, 0.35)	212.27 (175.93, 257.26)	187.74 (149.20, 236.20)	−0.42 (−0.67, −0.17)	0.13 (0.12, 0.13)	0.19 (0.18, 0.20)	1.58 (1.37, 1.80)	38.92 (27.11, 53.05)	37.81 (27.50, 50.48)	−0.59 (−0.70, −0.48)
North Africa and Middle East	3.52 (2.67, 4.64)	4.02 (3.00, 5.37)	0.51 (0.46, 0.57)	34.02 (26.74, 43.38)	39.52 (30.94, 50.54)	0.77 (0.60, 0.95)	0.09 (0.06, 0.15)	0.07 (0.05, 0.09)	−1.31 (−1.45, −1.17)	10.68 (7.78, 14.52)	9.92 (7.30, 13.27)	−0.60 (−0.70, −0.50)
Oceania	0.78 (0.56, 1.07)	0.91 (0.66, 1.24)	0.44 (0.39, 0.48)	6.75 (5.04, 9.00)	6.36 (4.68, 8.65)	−0.27 (−0.32, −0.22)	0.20 (0.10, 0.33)	0.12 (0.07, 0.20)	−1.99 (−2.18, −1.80)	11.64 (6.11, 18.33)	7.44 (4.70, 11.76)	−0.60 (−0.95, −0.25)
South Asia	5.29 (3.96, 7.06)	6.39 (4.81, 8.50)	0.74 (0.60, 0.88)	37.51 (28.81, 48.79)	44.21 (33.81, 57.81)	0.78 (0.61, 0.94)	0.18 (0.12, 0.27)	0.12 (0.08, 0.16)	−1.70 (−1.81, −1.58)	16.12 (11.91, 21.62)	13.48 (9.99, 17.93)	−0.64 (−0.80, −0.47)
Southeast Asia	0.71 (0.52, 0.96)	0.84 (0.62, 1.14)	0.54 (0.50, 0.57)	6.62 (5.07, 8.60)	6.72 (5.10, 8.84)	0.18 (0.13, 0.23)	0.08 (0.04, 0.10)	0.05 (0.03, 0.07)	−1.42 (−1.56, −1.29)	5.25 (3.40, 6.83)	4.05 (2.91, 5.16)	−0.81 (−0.99, −0.62)
Southern Latin America	5.55 (4.09, 7.56)	5.92 (4.39, 8.04)	0.21 (0.17, 0.25)	52.61 (40.27, 68.54)	55.50 (42.17, 73.75)	0.16 (0.14, 0.18)	0.13 (0.11, 0.14)	0.06 (0.06, 0.07)	−1.93 (−2.20, −1.67)	14.88 (11.24, 19.50)	11.92 (8.17, 17.12)	−0.97 (−1.19, −0.76)
Southern sub−Saharan Africa	1.80 (1.35, 2.38)	1.87 (1.41, 2.48)	0.21 (0.12, 0.30)	13.13 (10.09, 17.09)	15.03 (11.70, 19.43)	0.31 (0.27, 0.35)	0.14 (0.09, 0.19)	0.13 (0.09, 0.17)	−0.13 (−0.79, 0.53)	9.93 (7.01, 12.93)	9.15 (6.91, 11.83)	−1.01 (−1.11, −0.92)
Tropical Latin America	2.11 (1.58, 2.81)	3.12 (2.32, 4.21)	0.82 (0.20, 1.45)	14.24 (10.86, 18.51)	20.69 (15.55, 27.53)	0.86 (0.28, 1.44)	0.22 (0.21, 0.24)	0.22 (0.20, 0.23)	−0.13 (−0.42, 0.16)	14.13 (12.88, 15.64)	14.86 (13.30, 16.87)	−1.18 (−1.41, −0.94)
Western Europe	16.08 (13.03, 19.74)	17.13 (13.17, 22.01)	0.22 (0.02, 0.42)	173.43 (143.74, 208.22)	172.70 (137.31, 217.89)	−0.13 (−0.39, 0.13)	0.15 (0.14, 0.16)	0.14 (0.13, 0.15)	0.25 (−0.15, 0.65)	34.39 (24.93, 45.84)	33.37 (23.59, 45.35)	−1.57 (−1.93, −1.21)
Western sub−Saharan Africa	1.52 (1.14, 2.01)	1.64 (1.23, 2.16)	0.10 (−0.01, 0.20)	12.49 (9.68, 16.06)	14.64 (11.43, 18.81)	0.52 (0.49, 0.54)	0.56 (0.31, 0.77)	0.63 (0.32, 0.92)	0.38 (0.31, 0.46)	32.74 (18.85, 44.51)	37.11 (19.73, 53.42)	−1.80 (−1.98, −1.62)

### 3.2 Regional Patterns of IBD Burden

Among the five SDI regions, High‐SDI countries exhibited the highest ASIR (14.50, 95% UI: 11.22–18.44), ASPR (133.34, 95% UI: 106.01–167.48), and DALYs (26.59, 95% UI: 19.10–35.53), while Middle‐SDI countries had the lowest burden across these metrics. Interestingly, the highest ASMR was observed in Low‐SDI regions (0.26, 95% UI: 0.15–0.35), whereas the lowest was recorded in High‐Middle SDI regions (0.08, 95% UI: 0.07–0.09). Over time, incidence and prevalence increased across most SDI levels, especially in Middle‐SDI regions, where ASIR and ASPR showed the most rapid increases (EAPC = 1.65 and 1.44, respectively). In contrast, DALYs and mortality rates declined in most SDI regions, with the most significant reductions observed in High‐Middle SDI areas (EAPC for ASMR = −2.38; for DALYs = −1.13). Across the 21 GBD regions, Australasia reported the highest ASIR (24.82) and ASPR (217.01), whereas High‐Income North America showed the highest DALYs (37.81). Central Latin America had the lowest ASIR (0.66) and ASPR (5.84). The most substantial temporal increase in ASIR and ASPR was observed in East Asia (EAPC for ASIR = 3.06; for ASPR = 2.46). The greatest rise in ASMR and DALYs was found in Australasia (EAPC for ASMR = 2.65; for DALYs = 0.98) (Table 1).

### 3.3 Country‐Level IBD Burden

**FIGURE 1:**
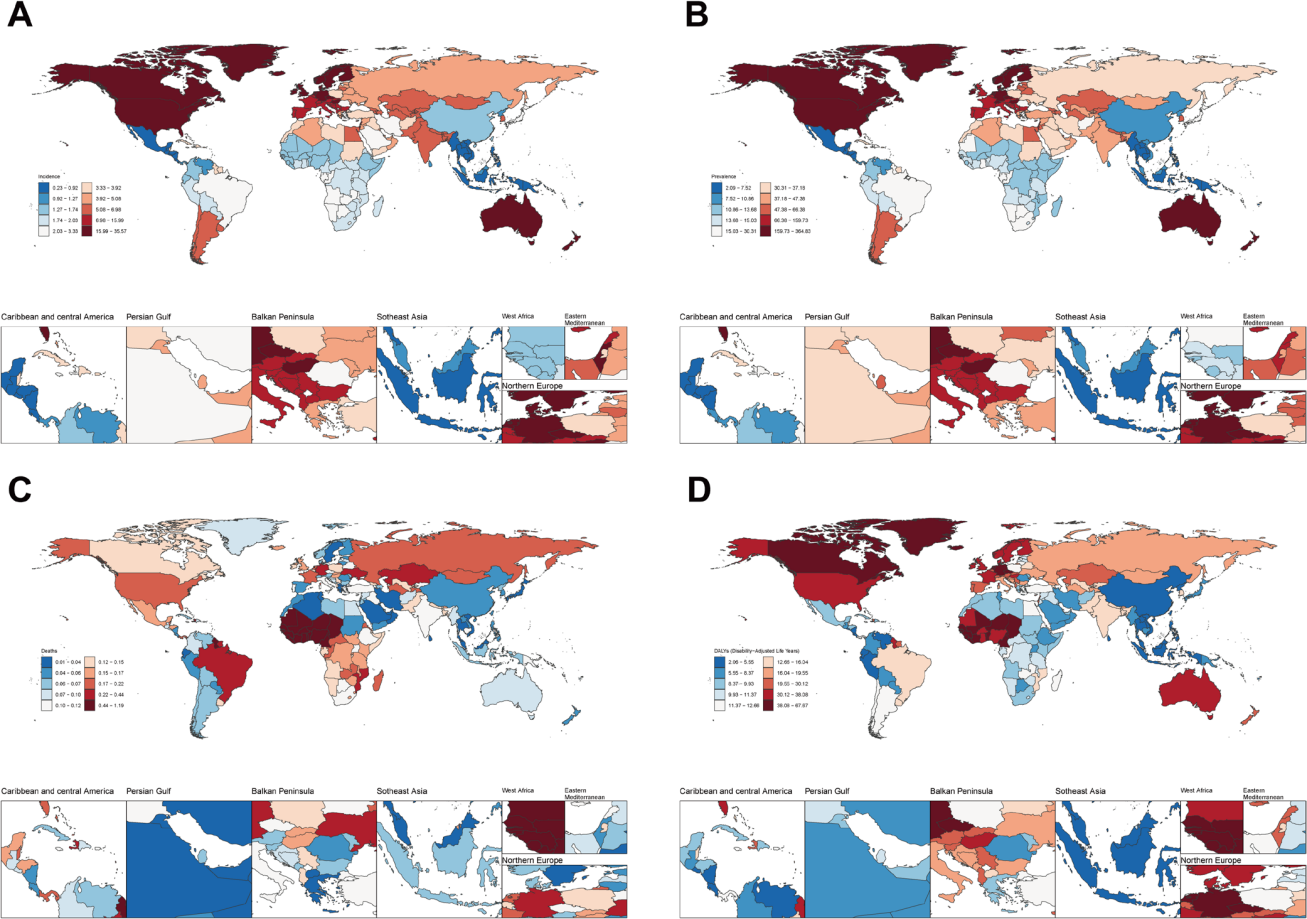
ASIR, ASPR, ASMR, and ASDR of IBD at the national level in 2021 (A, B, C, D).

At the national level, Canada had the highest ASIR (35.22) and ASPR (361.22) among the 15–49 age group in 2021. In contrast, Mexico reported the lowest values for both indicators (ASIR = 0.23; ASPR = 2.12). Guinea‐Bissau recorded the highest ASMR (1.18) and DALYs (67.00), while Singapore and Sri Lanka had the lowest (ASMR = 0.01; DALYs = 2.08) (Figure 1A–C). Longitudinal trends showed that China experienced the steepest increase in ASIR (EAPC = 3.12) and ASPR (EAPC = 2.52), while Australia and Libya had the most pronounced rises in ASMR (EAPC = 3.31) and DALYs (EAPC = 1.78), respectively. Complete data from 204 countries are presented in Table S1.

### 3.4 Age–Sex–Time Analysis

**FIGURE 2:**
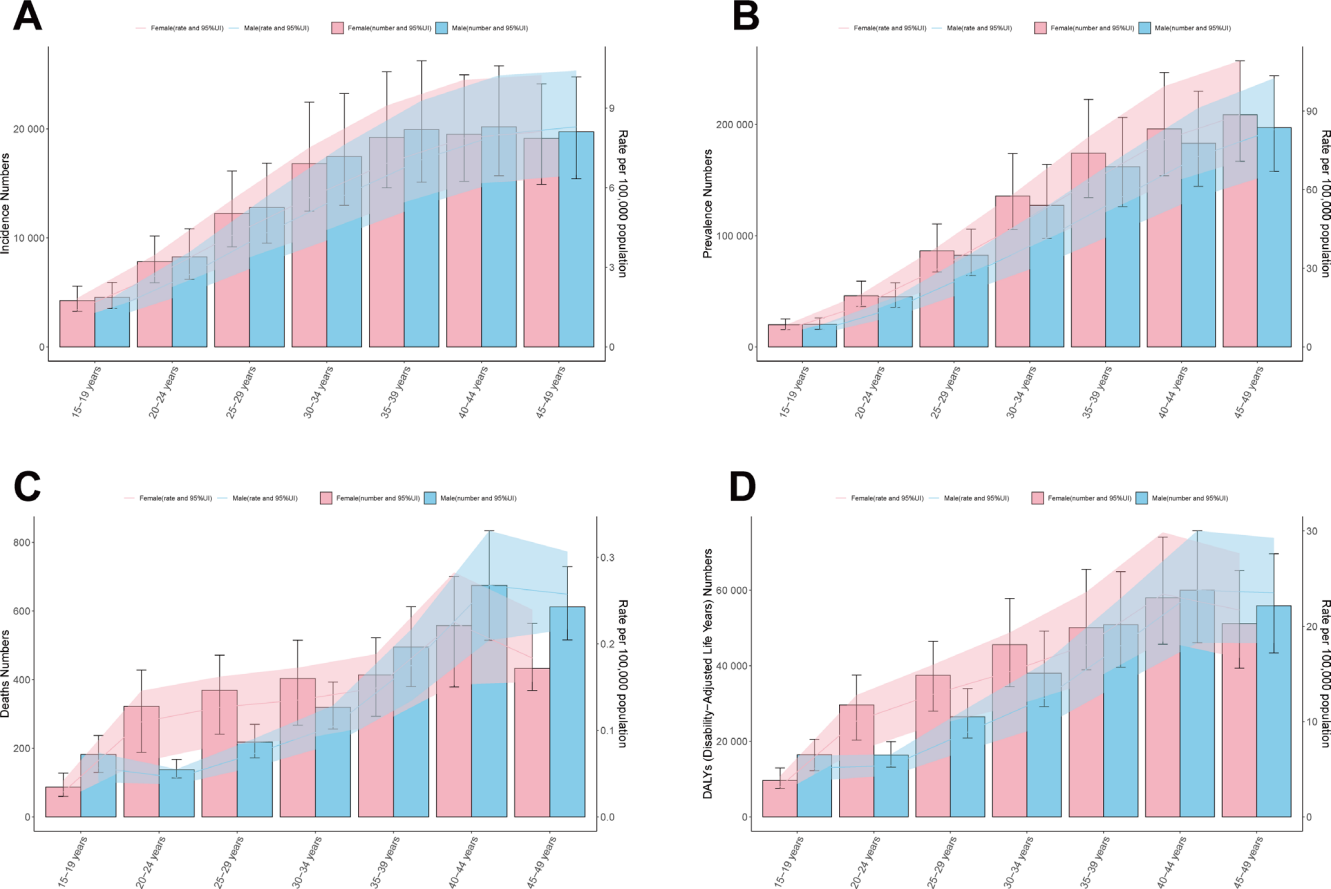
Sex‐ and age‐structured analysis of IBD disease burden in 2021. (A) Incident numbers and their corresponding rate; (B) Prevalent numbers and their corresponding rate; (C) Deaths and their mortality rate; (D) The numbers and rate of Disability‐Adjusted Life Years (DALYs).

Age‐specific analysis revealed a sharp increase in incidence and prevalence with age. In 2021, individuals aged 45–49 had nearly six times higher incidence and 13 times higher prevalence than those aged 15–19. No significant sex difference was observed in incidence or prevalence, but females aged 20–34 showed significantly higher mortality and DALYs than their male counterparts (Figure 2A–D). Temporal trends from 1990 to 2021 showed stable ASIR and ASPR across all age groups, with a peak around 2010. Notably, individuals aged 40–49 experienced significant declines in ASMR and DALYs. Overall, disease burden increased with age (Figure S6). Sex‐time analysis showed a “rise‐then‐decline” pattern in ASIR and ASPR from 1990 to 2021 in both sexes, while ASMR and DALYs showed overall downward trends with minimal sex differences (Figure S5).

### 3.5 Temporal Trends Based on Joinpoint Regression

**FIGURE 3:**
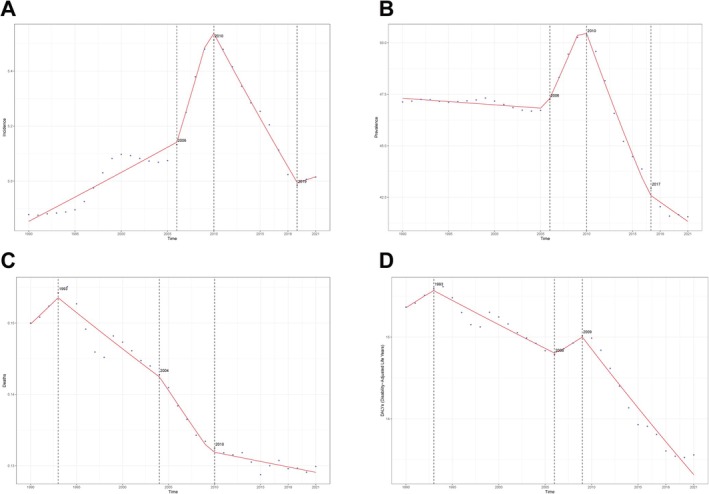
Trends in global IBD burden from 1990 to 2021. (A) ASIR, (B) ASPR, (C) ASMR, (D) ASDR.

Joinpoint regression analysis revealed that from 1990 to 2021, the global incidence of IBD among individuals aged 15–49 followed a trend of initial increase followed by a decline. The prevalence showed a pattern of a slight initial decrease, followed by a sharp rise, and then a significant decline. The death rate exhibited an initial increase, followed by a continuous decrease and eventual stabilization. DALYs displayed a trend of initial increase, subsequent decline, a brief rebound, and then a rapid decrease. Specifically, the AAPC for incidence was 0.005 (95% CI: 0.003, 0.007), for prevalence was −0.226 (95% CI: −0.253, −0.198), for mortality was −0.001 (95% CI: −0.001, −0.001), and for DALYs was −0.066 (95% CI: −0.074, −0.059). The years in which these four disease burden indicators experienced significant changes were concentrated between 2004 and 2010, as shown in Figure 3A–D.

### 3.6 Association Between IBD Burden and SDI

Globally and across GBD regions, SDI was positively correlated with ASIR and ASPR, negatively correlated with ASMR, and showed a non‐linear association with DALYs. As SDI increased, incidence and prevalence rose, while mortality declined. DALYs initially decreased and then showed a slow upward trend after an SDI of ~0.55. Australasia had the steepest rise in ASIR and ASPR (Figure 3A–C). Among 204 countries, ASIR (*p* = 0.000) and ASPR (*p* = 0.000) were positively associated with SDI, while ASMR was negatively correlated (*p* = 1.13e–10). The correlation between DALYs and SDI was weak (*r* = 0.1521), suggesting that while high‐SDI countries have higher absolute burdens, their rates of burden increase are slowing.

### 3.7 Age–Period–Cohort (APC) Analysis

The age–period–cohort (APC) analyses revealed both consistent and divergent patterns across incidence, prevalence, mortality, and DALYs. Age effects showed that all indicators increased with age: incidence and prevalence rose almost linearly from 15 to 49 years (Figures S1B, S2B), while mortality and DALYs reached a high plateau around age 40 (Figures S3B, S4B), indicating that age is a major determinant of IBD burden. Period effects demonstrated that incidence and prevalence showed a slight decline before 2005, followed by a sharp increase between 2005 and 2010 when they reached their peak, and then a sustained decline from 2010 to 2020 (Figures S1C, S2C). In contrast, mortality and DALYs declined steadily throughout the study period, reflecting improved diagnostic awareness, better treatment accessibility, and optimized long‐term management (Figures S3C, S4C). Cohort effects further highlighted heterogeneity: incidence displayed an inverted‐U pattern, peaking among cohorts born around 1980 (Figure S1D), while prevalence, mortality, and DALYs consistently declined in more recent birth cohorts, suggesting that younger generations have benefited from advances in public health and clinical care (Figures S2D, S3D, S4D). Overall, although age universally increases the IBD burden, period and cohort effects together have contributed to a gradual reduction in severe outcomes such as mortality and DALYs, whereas incidence and prevalence remain more sensitive to temporal and generational variations.

### 3.8 Forecast of IBD Burden in the 15–49‐Year‐Old Population

**FIGURE 4:**
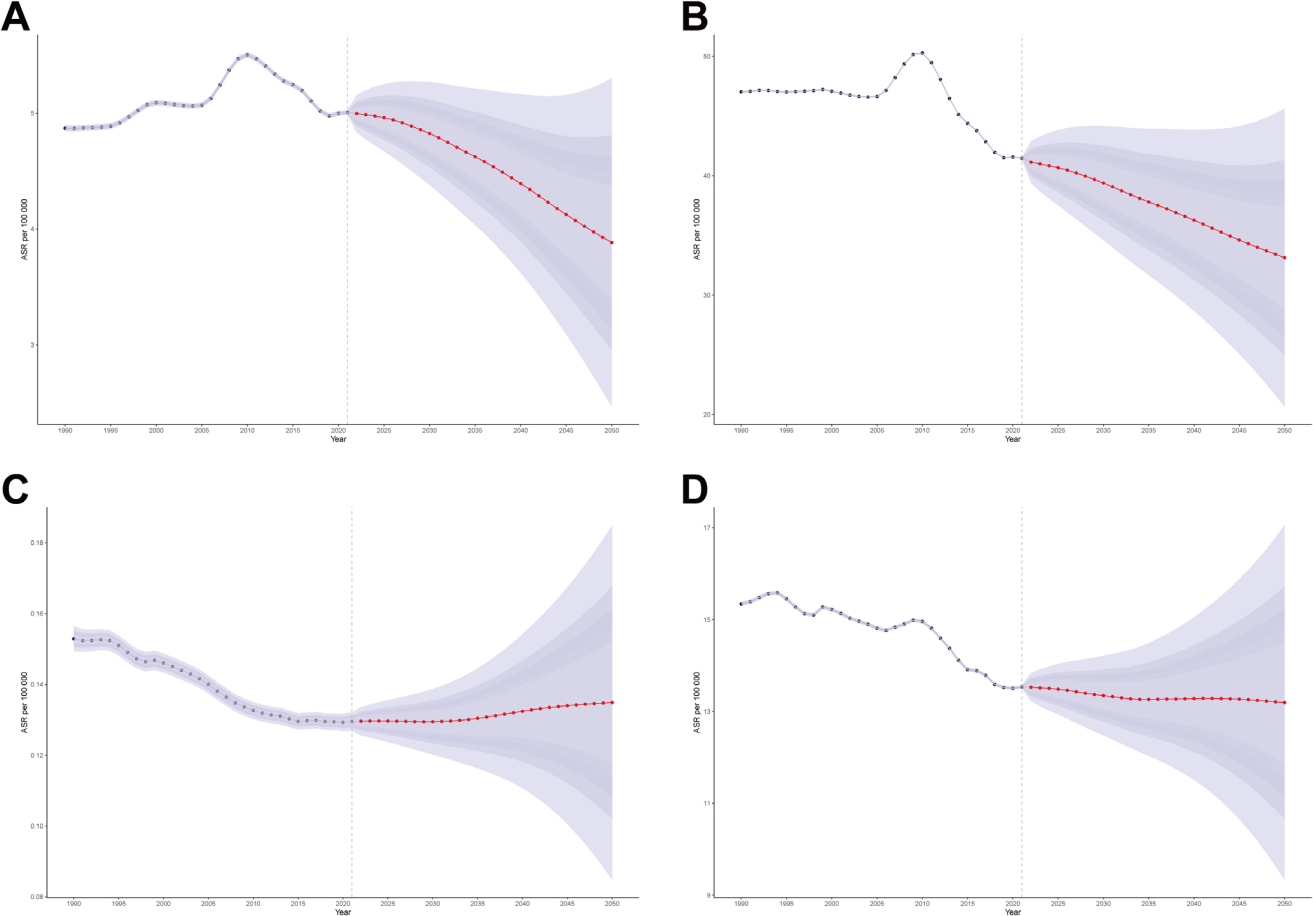
Change trend of ASR of inflammatory bowel disease in 15–49 from 1990 to 2050 (A) ASIR, (B) ASPR, (C) ASMR, (D) ASDR.

Projections from 2022 to 2050 suggest a gradual decline in IBD burden among individuals aged 15–49. Both ASIR and ASPR are expected to continue decreasing. ASMR is projected to rise slightly after 2020, though with considerable uncertainty. DALYs are projected to decline slowly and then stabilize. By 2050, the predicted ASIR and ASPR will decrease to 3.88 (95% CI: 2.47–5.30) and 33.14 (95% CI: 20.65–45.62) per 100,000, respectively, corresponding to approximately 173278.59 new cases and 1478466.47 prevalent cases (95% CI: 921393.34–2035539.59). Declines are expected across all age subgroups, with steeper reductions in older groups (Figure 4A–D).

## 4 Discussion

This study presents a comprehensive analysis of the global burden of inflammatory bowel disease (IBD) among individuals aged 15–49, using data from the Global Burden of Disease (GBD) 2021 study. We examined trends in incidence, prevalence, mortality, and disability‐adjusted life years (DALYs) across global, regional, and national levels. At the global level, we found that incidence has steadily increased—particularly in high SDI regions and East Asia—while overall mortality and DALYs have shown slight declines. However, substantial geographic and demographic disparities remain, and certain countries and regions even exhibited atypical upward trends. These findings indicate that the burden of IBD remains significant, and in some settings is worsening, underscoring the ongoing challenges in IBD prevention and management.

In 2021, IBD remained a major public health issue in this age group, with over 200,000 new cases, more than 1.6 million prevalent cases, and over 540,000 DALYs. Gender‐stratified analyses indicated that males had slightly higher incidence and mortality rates, while females showed marginally higher prevalence and DALYs—trends consistent with findings in the all‐age population [19]. These disparities may reflect differences in genetic heterogeneity, smoking, diet, and environmental sensitivity between sexes [20, 21]. Age‐specific analyses showed that incidence and prevalence peaked in the 45–49 age group, while mortality and DALYs were highest among those aged 40–44, possibly due to differences in screening rates and treatment responses. Notably, individuals aged 40–49 are typically at the peak of their careers and under substantial family responsibilities, making them not only a priority for clinical intervention but also a key target for public policy and health support systems [22].

Across the 21 GBD regions, the highest age‐standardized incidence rate (ASIR), prevalence rate (ASPR), and DALY rate (ASDR) were observed in High‐income North America, Western Europe, and Australasia, whereas the lowest values were found in Latin America, Oceania, Southeast Asia, and East Asia. Of note, Western Sub‐Saharan Africa exhibited unusually high ASDR. Age‐standardized mortality rates (ASMR) were highest in Western Sub‐Saharan Africa and lowest in High‐income Asia Pacific. At the national level, Canada and Mexico ranked first and last, respectively, in incidence and prevalence, while Guinea‐Bissau and Singapore had the highest and lowest mortality and DALYs. Correlation analysis confirmed a positive relationship between SDI and incidence/prevalence, and a negative association with mortality. These findings, consistent with trends observed across all age groups [19], highlight that the burden of IBD remains significant even in high‐SDI settings.

Originally considered a “Western disease”, IBD's global distribution is shaped by the timing and nature of industrialization [23]. Industrialized societies often face environmental and lifestyle changes—air pollution (e.g., PM2.5, NO_2_), water contamination, tobacco use [24], early antibiotic exposure [25], high intake of red meat and refined carbohydrates, low dietary fiber [26], unhealthy diets like high‐sugar or fat food [27] and obesity—all linked to increased IBD risk [28]. Additionally, enhanced healthcare access and health awareness in high‐income countries have led to improved detection [29]. These factors partly explain the dramatic increases in incidence and prevalence in middle‐ and low‐SDI regions such as China, where ASIR and ASPR showed the highest estimated annual percentage changes (EAPCs). Rapid urbanization, high hospitalization rates, increasing biologic therapy use, and unequal healthcare coverage have compounded the burden [30], presenting major challenges for developing health systems [31, 32]. Thus, context‐specific public health policies are urgently needed.

Despite an overall negative correlation between SDI and mortality/DALYs, we observed high EAPCs in ASMR and ASDR in Australasia and Libya. Although Australasia's ASMR remained below the global average during 1990–2021, its EAPC reached 2.65, suggesting rising mortality despite a low baseline burden [33, 34]. This may be attributed to a lack of multidisciplinary IBD care services in Australia and New Zealand [35], with surveys reporting widespread shortages of coordinated IBD teams [36, 37]. Without sustained medical support, patients face increased risks of complications and disease progression, contributing to rising mortality^20^. These findings indicate that even high‐income countries may face serious public health challenges if service development lags behind patient demand.

Joinpoint regression analysis suggests that the global epidemiology of IBD has undergone notable changes. Since the beginning of the new century, both the incidence and prevalence have shown a pattern of “a sharp rise followed by a sharp decline” after 2006. Projections suggest that by 2025, incidence and prevalence will decline slightly, mortality may increase marginally, and DALYs will stabilize. These trends likely reflect combined influences of global economic and environmental changes, advancements in medical technology, and improved individual health practices. Environmental and lifestyle shifts associated with industrialization affect gut barrier function, microbiota homeostasis, inflammatory responses, and genetic susceptibility [24]. Moreover, improved health awareness and novel therapies (e.g., biologics, fecal microbiota transplantation) have positively influenced disease control [38, 39, 40]. Nevertheless, as a chronic, treatment‐resistant condition, IBD continues to impose a heavy burden on patients and their families, particularly in the working‐age population, and poses a formidable challenge to healthcare systems worldwide. Strengthening early screening programs, healthcare workforce training, and public education efforts is essential to alleviating the global IBD burden.

This study has several important limitations. First, the findings are based on GBD data, which are generated through statistical modeling and multi‐source estimation rather than systematic field investigations or clinical registry data. The accuracy of the results may be influenced by data quality, modeling assumptions, and estimation methods—especially in resource‐limited settings where data gaps and underdiagnosis are common. Second, due to the lack of standardized subtype classification, GBD does not provide separate epidemiological data for ulcerative colitis (UC) and Crohn's disease (CD), limiting deeper analysis of IBD heterogeneity and risk factors. Future studies should incorporate robust field surveys and registry data, promote standardized classification of IBD subtypes, and comprehensively assess disparities in disease burden across populations and regions.

## 5 Conclusion

IBD remains a major health burden among people aged 15–49, with notable disparities across regions, genders, and age groups. The rising incidence in East Asia and sustained burden in high‐SDI areas highlight the need for targeted strategies. Improving early detection, access to care, and health system responses will be key to reducing the global impact of IBD.

## Conflicts of Interest

The authors declare no conflicts of interest.

## Supporting information


**Table S1:** EAPCs of age‐standardized rates (incidence, prevalence, mortality, and DALYs) in 204 countries worldwide.
**Figure S1:** Results of the age‐period‐cohort analysis of incidence (A. Net drifts and local drifts of incidence; B. Age effects on incidence; C. Period effects on incidence; D. Cohort effects on incidence).
**Figure S2:** Results of the age‐period‐cohort analysis of prevalence (A. Net drifts and local drifts of prevalence; B. Age effects on prevalence; C. Period effects on prevalence; D. Cohort effects on prevalence).
**Figure S3:** Results of the age‐period‐cohort analysis of mortality (A. Net drifts and local drifts of mortality; B. Age effects on mortality; C. Period effects on mortality; D. Cohort effects on mortality).
**Figure S4:** Results of the age‐period‐cohort analysis of DALYs (A. Net drifts and local drifts of DALYs; B. Age effects on DALYs; C. Period effects on DALYs; D. Cohort effects on DALYs).
**Figure S5:** Global trends in age‐standardized rates of IBD from 1990 to 2021, stratified by sex (A. Incidence; B. Prevalence; C. Mortality; D. DALYs).
**Figure S6:** Global temporal trends in age‐specific rates of IBD from 1990 to 2021 (A. Incidence; B. Prevalence; C. Mortality; D. DALYs).

**Data S1:** Supporting Information.

## Data Availability

The data that support the findings of this study are available from the corresponding author upon reasonable request.
