# Supplementary material for: Global Trends and Future Projections in the Burden of Inflammatory Bowel Disease Among Adolescents and Young Adults (15–49 Years) From 1990 to 2021
Source: JGH Open. 2025 Sep 18;9(9):e70282. doi: 10.1002/jgh3.70282 (PMC12446572; doi:10.1002/jgh3.70282)

**Supplementary Table1**

EAPCs of Age-Standardized Rates (Incidence, Prevalence, Mortality, and DALYs) in 204 Countries Worldwide

| Location | EAPC of ASIR | EAPC of ASPR | EAPC of ASMR | EAPC of ASDR |
| --- | --- | --- | --- | --- |
| Afghanistan | 0.54(0.47,0.61) | 0.77(0.64,0.90) | 0.15(-0.06,0.37) | 0.40(0.23,0.57) |
| Albania | 0.92(0.82,1.02) | 0.03(-0.04,0.10) | -1.45(-1.69,-1.21) | -0.77(-0.88,-0.67) |
| Algeria | 0.75(0.68,0.83) | 0.83(0.68,0.99) | -0.18(-0.34,-0.02) | 0.47(0.34,0.60) |
| American Samoa | 0.43(0.39,0.48) | -0.04(-0.08,-0.01) | -5.17(-5.77,-4.56) | -4.82(-5.39,-4.25) |
| Andorra | 0.18(0.14,0.22) | 0.18(0.17,0.19) | -0.49(-0.71,-0.28) | 0.07(0.04,0.11) |
| Angola | 0.84(0.78,0.89) | 0.42(0.35,0.49) | 0.10(-0.08,0.27) | 0.21(0.06,0.35) |
| Antigua and Barbuda | 0.52(0.46,0.58) | 0.19(0.19,0.20) | -3.48(-3.86,-3.11) | -2.30(-2.57,-2.03) |
| Argentina | 0.08(0.05,0.12) | 0.13(0.10,0.16) | -1.56(-1.81,-1.31) | -0.43(-0.51,-0.34) |
| Armenia | 0.72(0.67,0.76) | 0.44(0.41,0.47) | -1.40(-1.88,-0.92) | -0.56(-0.83,-0.29) |
| Australia | 0.82(0.45,1.19) | 0.98(0.41,1.56) | 3.31(2.32,4.31) | 1.16(0.61,1.71) |
| Austria | 0.24(0.21,0.28) | -0.03(-0.19,0.13) | -0.69(-1.15,-0.24) | -0.15(-0.31,0.00) |
| Azerbaijan | 0.23(0.18,0.29) | 0.35(0.25,0.45) | -1.23(-1.48,-0.98) | -0.23(-0.38,-0.08) |
| Bahamas | 0.73(0.63,0.83) | -0.03(-0.05,-0.01) | -2.89(-3.18,-2.61) | -2.10(-2.30,-1.90) |
| Bahrain | 0.88(0.73,1.03) | 0.64(0.49,0.80) | -0.41(-0.48,-0.34) | 0.28(0.16,0.39) |
| Bangladesh | 0.28(0.20,0.36) | 0.64(0.59,0.70) | -1.24(-1.34,-1.14) | -0.31(-0.38,-0.24) |
| Barbados | 0.40(0.24,0.57) | 0.04(-0.14,0.23) | -1.26(-1.52,-1.00) | -0.78(-0.99,-0.57) |
| Belarus | 0.82(0.78,0.87) | 0.39(0.29,0.48) | -0.57(-1.10,-0.05) | -0.10(-0.33,0.12) |
| Belgium | 0.94(0.77,1.10) | 0.19(0.07,0.31) | -0.01(-0.60,0.59) | 0.10(-0.06,0.26) |
| Belize | 0.70(0.63,0.77) | 0.18(0.15,0.20) | -1.93(-2.37,-1.49) | -1.45(-1.79,-1.11) |
| Benin | -0.11(-0.20,-0.01) | 0.69(0.66,0.73) | 0.20(0.06,0.34) | 0.26(0.13,0.39) |
| Bermuda | 0.54(0.49,0.60) | 0.20(0.18,0.21) | -5.42(-6.07,-4.77) | -3.40(-3.90,-2.90) |
| Bhutan | 0.86(0.79,0.94) | 0.68(0.61,0.75) | -1.15(-1.22,-1.08) | -0.33(-0.38,-0.28) |
| Bolivia (Plurinational State of) | 0.50(0.41,0.59) | 0.16(0.09,0.23) | -1.05(-1.17,-0.93) | -0.69(-0.78,-0.59) |
| Bosnia and Herzegovina | 0.99(0.76,1.21) | 0.33(0.23,0.43) | -1.63(-1.79,-1.47) | -0.55(-0.63,-0.46) |
| Botswana | 0.76(0.71,0.81) | 0.47(0.43,0.52) | -1.44(-1.69,-1.19) | -0.98(-1.17,-0.79) |
| Brazil | 0.84(0.20,1.48) | 0.89(0.30,1.48) | -0.12(-0.42,0.17) | 0.13(-0.10,0.36) |
| Brunei Darussalam | 0.23(0.19,0.27) | 0.16(0.13,0.19) | -0.42(-0.65,-0.19) | -0.41(-0.59,-0.23) |
| Bulgaria | 0.70(0.63,0.78) | 0.07(0.03,0.12) | 0.50(0.10,0.91) | 0.17(0.07,0.27) |
| Burkina Faso | -0.08(-0.12,-0.03) | 0.65(0.62,0.67) | 0.51(0.23,0.78) | 0.54(0.28,0.80) |
| Burundi | 0.53(0.46,0.60) | -0.11(-0.20,-0.01) | -0.44(-0.53,-0.34) | -0.36(-0.46,-0.26) |
| Cabo Verde | 0.38(0.30,0.46) | 0.59(0.57,0.60) | -2.21(-2.28,-2.14) | -1.97(-2.04,-1.91) |
| Cambodia | 0.78(0.75,0.81) | 0.12(0.07,0.16) | -0.82(-0.92,-0.72) | -0.73(-0.82,-0.65) |
| Cameroon | 0.29(0.15,0.42) | 0.35(0.28,0.42) | -0.06(-0.15,0.04) | 0.01(-0.08,0.09) |
| Canada | -0.54(-0.88,-0.20) | -1.20(-1.70,-0.69) | 0.91(0.67,1.16) | -1.09(-1.57,-0.62) |
| Central African Republic | 0.29(0.28,0.30) | 0.01(-0.03,0.05) | 0.40(0.30,0.50) | 0.32(0.25,0.40) |
| Chad | -0.10(-0.21,0.01) | 0.56(0.53,0.59) | 0.97(0.84,1.10) | 0.98(0.86,1.11) |
| Chile | 0.40(0.30,0.50) | 0.24(0.22,0.26) | -2.78(-3.21,-2.35) | -0.97(-1.17,-0.76) |
| China | 3.12(2.44,3.80) | 2.52(1.74,3.30) | -2.82(-3.04,-2.61) | -1.17(-1.41,-0.93) |
| Colombia | 0.33(0.09,0.58) | 0.08(-0.25,0.41) | -0.37(-0.71,-0.04) | -0.24(-0.44,-0.05) |
| Comoros | 0.61(0.44,0.79) | 0.22(0.13,0.31) | -0.69(-1.09,-0.30) | -0.52(-0.83,-0.20) |
| Congo | 0.77(0.72,0.82) | 0.22(0.14,0.30) | 0.36(0.19,0.53) | 0.34(0.21,0.46) |
| Cook Islands | 0.69(0.64,0.75) | 0.01(-0.02,0.04) | -2.17(-2.41,-1.92) | -2.07(-2.32,-1.83) |
| Costa Rica | -0.01(-0.07,0.05) | 0.03(0.01,0.06) | 0.95(0.59,1.30) | 0.64(0.40,0.88) |
| Cote d'Ivoire | 0.06(-0.08,0.20) | 0.27(0.21,0.34) | 0.90(0.77,1.04) | 0.90(0.77,1.03) |
| Croatia | 0.60(0.02,1.18) | 0.07(-0.59,0.73) | -1.10(-1.47,-0.74) | -0.27(-0.78,0.24) |
| Cuba | 0.38(0.35,0.41) | 0.05(0.01,0.09) | -3.94(-4.33,-3.54) | -2.03(-2.29,-1.77) |
| Cyprus | 0.18(-0.00,0.36) | 0.55(0.50,0.61) | -1.92(-2.06,-1.77) | -0.29(-0.35,-0.23) |
| Czechia | 1.03(0.66,1.40) | 0.92(0.38,1.46) | -1.05(-1.40,-0.70) | 0.60(0.10,1.10) |
| Democratic People's Republic of Korea | 0.53(0.49,0.58) | -0.16(-0.22,-0.09) | -0.72(-0.80,-0.65) | -0.64(-0.70,-0.57) |
| Democratic Republic of the Congo | 0.97(0.94,1.00) | -0.48(-0.67,-0.29) | 0.18(0.04,0.32) | 0.00(-0.15,0.15) |
| Denmark | -0.65(-0.97,-0.32) | -0.47(-0.92,-0.02) | -0.81(-1.33,-0.30) | -0.50(-0.96,-0.05) |
| Djibouti | 0.36(0.29,0.42) | 0.28(0.22,0.35) | -0.67(-0.88,-0.46) | -0.45(-0.62,-0.29) |
| Dominica | 0.53(0.39,0.67) | 0.27(0.22,0.32) | -0.41(-0.59,-0.23) | -0.22(-0.37,-0.08) |
| Dominican Republic | 0.64(0.57,0.72) | 0.17(0.10,0.25) | -0.67(-1.00,-0.35) | -0.32(-0.52,-0.12) |
| Ecuador | 0.25(0.18,0.31) | 0.24(0.16,0.32) | -1.51(-2.03,-0.98) | -0.81(-1.13,-0.49) |
| Egypt | 0.67(0.60,0.75) | 0.80(0.62,0.97) | -1.80(-2.39,-1.20) | -0.39(-0.59,-0.18) |
| El Salvador | -0.40(-0.44,-0.36) | 0.69(0.62,0.75) | -0.56(-0.83,-0.29) | -0.38(-0.60,-0.15) |
| Equatorial Guinea | 1.29(1.06,1.51) | 1.19(0.99,1.39) | -1.35(-1.48,-1.22) | -0.84(-0.97,-0.72) |
| Eritrea | 0.79(0.73,0.86) | -0.00(-0.08,0.08) | -0.13(-0.25,-0.01) | -0.10(-0.21,0.02) |
| Estonia | 1.09(0.93,1.25) | 0.41(0.29,0.53) | -8.22(-9.86,-6.56) | -3.87(-4.84,-2.89) |
| Eswatini | 0.42(0.37,0.48) | 0.52(0.48,0.55) | 0.29(-0.07,0.66) | 0.35(0.05,0.65) |
| Ethiopia | 0.83(0.73,0.92) | 0.25(0.10,0.40) | -0.53(-0.65,-0.40) | -0.35(-0.46,-0.24) |
| Fiji | 0.79(0.68,0.89) | -0.09(-0.12,-0.06) | -1.42(-1.58,-1.25) | -1.18(-1.31,-1.04) |
| Finland | -1.40(-1.85,-0.96) | -1.19(-1.75,-0.63) | 0.05(-0.53,0.64) | -1.10(-1.61,-0.59) |
| France | -0.20(-0.41,0.01) | -0.61(-0.88,-0.34) | 0.76(0.33,1.19) | -0.27(-0.49,-0.05) |
| Gabon | 0.67(0.64,0.70) | 0.17(0.12,0.23) | 0.12(-0.01,0.25) | 0.13(0.03,0.22) |
| Gambia | 0.51(0.41,0.62) | 0.27(0.24,0.31) | 0.61(0.34,0.88) | 0.65(0.39,0.91) |
| Georgia | 0.22(0.17,0.26) | 0.12(0.06,0.19) | -1.90(-2.93,-0.86) | -0.97(-1.47,-0.47) |
| Germany | 0.80(0.40,1.21) | 0.65(0.21,1.10) | 0.84(0.47,1.20) | 0.63(0.23,1.02) |
| Ghana | 0.38(0.27,0.50) | 0.42(0.39,0.45) | -0.05(-0.13,0.03) | -0.04(-0.11,0.04) |
| Global | 0.21(0.09,0.34) | -0.32(-0.49,-0.14) | -0.65(-0.72,-0.59) | -0.45(-0.52,-0.38) |
| Greece | 0.86(0.35,1.36) | 0.48(-0.10,1.07) | 1.93(1.39,2.46) | 0.69(0.17,1.23) |
| Greenland | 0.51(0.46,0.55) | -0.12(-0.15,-0.09) | -0.25(-0.37,-0.12) | -0.16(-0.20,-0.13) |
| Grenada | 0.82(0.76,0.87) | 0.33(0.31,0.34) | -2.62(-2.89,-2.36) | -2.24(-2.44,-2.04) |
| Guam | 0.65(0.59,0.70) | -0.02(-0.05,0.01) | -2.26(-2.73,-1.79) | -1.54(-1.86,-1.23) |
| Guatemala | 0.20(0.12,0.28) | 0.32(0.29,0.35) | -0.64(-1.06,-0.22) | -0.55(-0.92,-0.17) |
| Guinea | -0.09(-0.20,0.02) | 0.55(0.51,0.59) | 0.75(0.62,0.89) | 0.78(0.65,0.90) |
| Guinea-Bissau | -0.05(-0.17,0.07) | 0.53(0.51,0.55) | 0.70(0.54,0.86) | 0.72(0.56,0.87) |
| Guyana | 0.71(0.65,0.77) | -0.00(-0.10,0.09) | -0.52(-0.86,-0.17) | -0.49(-0.80,-0.18) |
| Haiti | 0.50(0.42,0.58) | 0.16(0.10,0.23) | -0.35(-0.43,-0.27) | -0.26(-0.35,-0.18) |
| Honduras | 0.12(0.05,0.19) | 0.28(0.26,0.31) | -1.31(-1.40,-1.23) | -1.24(-1.33,-1.15) |
| Hungary | 0.46(0.05,0.87) | -0.31(-0.70,0.08) | -1.78(-2.11,-1.45) | -0.68(-1.01,-0.34) |
| Iceland | -0.70(-0.84,-0.56) | -1.08(-1.24,-0.92) | 1.07(0.70,1.44) | -0.73(-0.88,-0.58) |
| India | 0.81(0.64,0.99) | 0.87(0.66,1.08) | -1.86(-2.02,-1.70) | -0.64(-0.79,-0.49) |
| Indonesia | 0.24(0.19,0.28) | -0.15(-0.18,-0.12) | -1.72(-1.82,-1.62) | -1.36(-1.43,-1.28) |
| Iran (Islamic Republic of) | 0.23(-0.09,0.56) | 0.43(0.08,0.78) | 0.24(-0.18,0.65) | 0.35(0.11,0.60) |
| Iraq | 1.13(1.07,1.20) | 1.04(0.96,1.12) | -1.05(-1.16,-0.93) | -0.28(-0.36,-0.21) |
| Ireland | 0.08(-0.03,0.20) | 0.37(0.24,0.50) | 0.60(-0.01,1.21) | 0.39(0.16,0.62) |
| Israel | -0.09(-0.61,0.45) | -0.56(-1.30,0.18) | 0.60(-0.05,1.25) | -0.47(-1.17,0.24) |
| Italy | -0.63(-0.80,-0.47) | -1.02(-1.29,-0.76) | 1.10(0.70,1.50) | -0.38(-0.59,-0.17) |
| Jamaica | 0.33(0.23,0.43) | 0.21(0.20,0.22) | -0.58(-1.13,-0.03) | -0.28(-0.58,0.02) |
| Japan | -0.12(-0.68,0.43) | -0.28(-0.90,0.35) | -1.98(-2.16,-1.80) | -0.89(-1.30,-0.48) |
| Jordan | 0.76(0.59,0.93) | 0.69(0.50,0.88) | -0.41(-0.64,-0.18) | 0.23(0.08,0.38) |
| Kazakhstan | 0.64(0.51,0.76) | 0.25(0.20,0.30) | -0.74(-1.29,-0.20) | -0.45(-0.82,-0.07) |
| Kenya | 0.80(0.72,0.89) | 0.24(0.19,0.29) | 0.10(-0.03,0.24) | 0.12(0.02,0.23) |
| Kiribati | 0.54(0.50,0.57) | -0.08(-0.10,-0.05) | -1.60(-1.72,-1.47) | -1.50(-1.62,-1.38) |
| Kuwait | -0.61(-1.12,-0.10) | -0.78(-1.27,-0.29) | -2.64(-4.66,-0.58) | -1.33(-2.14,-0.51) |
| Kyrgyzstan | 0.22(0.20,0.24) | 0.11(0.04,0.18) | -5.62(-6.76,-4.48) | -3.31(-3.99,-2.63) |
| Lao People's Democratic Republic | 0.71(0.67,0.74) | 0.44(0.37,0.51) | -1.12(-1.21,-1.02) | -0.69(-0.77,-0.62) |
| Latvia | 0.73(0.65,0.81) | 0.32(0.24,0.39) | -6.88(-8.66,-5.06) | -3.60(-4.65,-2.54) |
| Lebanon | 0.77(0.72,0.82) | 0.62(0.48,0.76) | -0.94(-1.12,-0.77) | 0.08(0.02,0.14) |
| Lesotho | 0.41(0.34,0.47) | 0.51(0.48,0.54) | 1.23(0.85,1.60) | 1.09(0.80,1.39) |
| Liberia | -0.21(-0.42,-0.01) | 0.64(0.58,0.71) | 0.50(0.33,0.67) | 0.54(0.38,0.69) |
| Libya | 2.52(2.37,2.68) | 1.94(1.62,2.27) | 1.74(1.21,2.27) | 1.78(1.56,1.99) |
| Lithuania | 1.35(0.81,1.90) | 0.88(0.21,1.56) | -5.96(-7.66,-4.22) | -2.09(-2.87,-1.30) |
| Luxembourg | -0.22(-0.30,-0.15) | 0.33(0.28,0.37) | -1.31(-1.84,-0.78) | -0.05(-0.17,0.07) |
| Madagascar | 0.66(0.58,0.73) | 0.04(-0.10,0.17) | 0.03(-0.12,0.18) | 0.05(-0.07,0.18) |
| Malawi | 0.92(0.84,1.01) | 0.38(0.34,0.43) | 0.13(0.01,0.25) | 0.18(0.07,0.29) |
| Malaysia | 1.55(1.26,1.85) | 1.44(1.11,1.78) | -1.84(-2.14,-1.54) | 0.39(0.12,0.66) |
| Maldives | 0.98(0.86,1.09) | 0.64(0.58,0.70) | -0.80(-0.95,-0.65) | -0.27(-0.35,-0.18) |
| Mali | -0.07(-0.16,0.03) | 0.65(0.60,0.70) | 0.48(0.42,0.53) | 0.53(0.47,0.58) |
| Malta | -0.13(-0.22,-0.05) | 0.00(-0.04,0.04) | 1.50(0.98,2.03) | 0.33(0.22,0.44) |
| Marshall Islands | 0.55(0.52,0.58) | -0.06(-0.08,-0.03) | -2.13(-2.22,-2.04) | -2.01(-2.09,-1.92) |
| Mauritania | -0.10(-0.32,0.13) | 0.52(0.47,0.58) | 0.19(0.05,0.32) | 0.23(0.10,0.36) |
| Mauritius | 0.50(0.47,0.53) | 0.32(0.29,0.36) | 1.90(0.30,3.52) | 1.48(0.34,2.63) |
| Mexico | 0.04(-0.27,0.35) | 0.14(-0.10,0.38) | 1.50(1.11,1.89) | 1.45(1.07,1.82) |
| Micronesia (Federated States of) | 0.57(0.53,0.61) | -0.16(-0.20,-0.13) | -1.96(-2.12,-1.81) | -1.86(-2.00,-1.71) |
| Monaco | -0.11(-0.16,-0.06) | 0.14(0.14,0.15) | -0.08(-0.22,0.06) | 0.10(0.08,0.12) |
| Mongolia | 0.68(0.65,0.70) | 0.13(0.11,0.16) | -1.48(-1.64,-1.33) | -0.93(-1.02,-0.83) |
| Montenegro | 0.31(0.24,0.39) | 0.21(0.18,0.24) | -0.54(-0.79,-0.29) | -0.02(-0.08,0.04) |
| Morocco | 0.75(0.70,0.79) | 0.88(0.78,0.97) | 0.03(-0.16,0.22) | 0.51(0.40,0.62) |
| Mozambique | 0.85(0.80,0.90) | 0.28(0.22,0.34) | 1.31(1.20,1.42) | 1.17(1.09,1.25) |
| Myanmar | 0.83(0.77,0.88) | 0.51(0.43,0.58) | -1.55(-1.71,-1.38) | -1.21(-1.34,-1.07) |
| Namibia | 0.22(0.12,0.31) | 0.11(0.05,0.17) | -0.73(-0.89,-0.57) | -0.61(-0.73,-0.48) |
| Nauru | 0.42(0.30,0.53) | -0.12(-0.20,-0.04) | -1.87(-2.04,-1.70) | -1.74(-1.90,-1.58) |
| Nepal | 0.76(0.66,0.86) | 0.33(0.29,0.37) | -1.68(-1.79,-1.58) | -0.84(-0.92,-0.76) |
| Netherlands | 1.22(0.53,1.91) | 0.29(-0.45,1.02) | -0.11(-0.56,0.34) | 0.18(-0.48,0.83) |
| New Zealand | 0.09(-0.06,0.24) | -0.05(-0.24,0.14) | -1.15(-2.04,-0.25) | -0.14(-0.38,0.10) |
| Nicaragua | 0.05(-0.08,0.18) | 0.21(0.14,0.28) | -0.07(-0.29,0.15) | -0.02(-0.18,0.15) |
| Niger | -0.17(-0.25,-0.08) | 0.30(0.28,0.33) | 0.28(0.16,0.41) | 0.30(0.17,0.42) |
| Nigeria | 0.06(-0.04,0.17) | 0.58(0.55,0.62) | 0.35(0.24,0.45) | 0.41(0.31,0.51) |
| Niue | 0.78(0.72,0.84) | -0.09(-0.12,-0.06) | -2.11(-2.22,-2.00) | -1.94(-2.05,-1.84) |
| North Macedonia | 0.73(0.64,0.82) | 0.16(0.13,0.19) | -0.98(-1.24,-0.72) | -0.08(-0.12,-0.05) |
| Northern Mariana Islands | 0.40(0.34,0.45) | -0.24(-0.28,-0.20) | -4.40(-4.85,-3.95) | -4.01(-4.45,-3.57) |
| Norway | 0.50(0.40,0.60) | 0.10(0.04,0.16) | -3.19(-3.83,-2.54) | -0.44(-0.57,-0.31) |
| Oman | 1.22(1.12,1.31) | 1.08(0.98,1.18) | 0.33(0.02,0.65) | 0.79(0.67,0.92) |
| Pakistan | 0.63(0.55,0.72) | 0.31(0.23,0.40) | -0.97(-1.13,-0.81) | -0.41(-0.50,-0.32) |
| Palau | 0.50(0.45,0.55) | -0.22(-0.25,-0.19) | -0.66(-0.83,-0.49) | -0.56(-0.72,-0.39) |
| Palestine | 1.18(1.13,1.23) | 0.86(0.76,0.96) | -2.68(-2.97,-2.39) | -1.39(-1.62,-1.15) |
| Panama | 0.11(0.07,0.14) | 0.17(0.14,0.19) | 1.13(0.82,1.43) | 0.98(0.72,1.25) |
| Papua New Guinea | 0.48(0.45,0.51) | -0.22(-0.27,-0.16) | -1.80(-2.04,-1.56) | -1.62(-1.85,-1.40) |
| Paraguay | 0.08(-0.10,0.25) | -0.29(-0.51,-0.08) | 0.34(0.19,0.50) | 0.01(-0.13,0.16) |
| Peru | 0.62(0.53,0.72) | -0.09(-0.23,0.06) | -1.66(-1.99,-1.32) | -1.13(-1.41,-0.85) |
| Philippines | 0.45(0.40,0.49) | -0.38(-0.40,-0.35) | -1.68(-2.00,-1.37) | -1.45(-1.69,-1.20) |
| Poland | 0.36(0.23,0.50) | 0.08(-0.01,0.17) | -0.68(-0.91,-0.45) | -0.36(-0.52,-0.21) |
| Portugal | 0.79(0.43,1.14) | 0.50(0.16,0.84) | -0.60(-1.13,-0.07) | 0.03(-0.33,0.40) |
| Puerto Rico | 0.59(0.43,0.75) | 0.12(-0.07,0.32) | -4.37(-4.73,-4.01) | -2.95(-3.19,-2.70) |
| Qatar | 0.77(0.75,0.79) | 0.76(0.69,0.82) | -1.83(-2.08,-1.58) | -0.47(-0.58,-0.36) |
| Republic of Korea | 2.08(1.23,2.92) | 2.09(1.20,2.98) | -4.73(-5.01,-4.45) | -0.55(-1.02,-0.07) |
| Republic of Moldova | 0.58(0.50,0.66) | 0.34(0.18,0.50) | -6.27(-7.84,-4.68) | -3.30(-4.28,-2.32) |
| Romania | 1.45(0.55,2.36) | 0.69(-0.14,1.53) | -0.71(-0.91,-0.50) | 0.01(-0.50,0.52) |
| Russian Federation | 0.49(0.46,0.51) | 0.16(0.03,0.29) | -2.51(-3.10,-1.92) | -1.82(-2.28,-1.36) |
| Rwanda | 0.91(0.82,1.00) | 0.15(0.07,0.23) | -0.69(-0.92,-0.46) | -0.53(-0.73,-0.33) |
| Saint Kitts and Nevis | 0.52(0.40,0.63) | 0.24(0.15,0.32) | -5.01(-5.79,-4.22) | -4.00(-4.65,-3.35) |
| Saint Lucia | 0.40(0.35,0.44) | 0.25(0.21,0.29) | -2.61(-3.06,-2.15) | -1.92(-2.26,-1.57) |
| Saint Vincent and the Grenadines | 0.52(0.45,0.59) | 0.20(0.16,0.24) | -1.56(-1.85,-1.26) | -1.39(-1.65,-1.12) |
| Samoa | 0.48(0.44,0.53) | -0.07(-0.11,-0.04) | -1.49(-1.64,-1.34) | -1.39(-1.53,-1.25) |
| San Marino | 0.08(-0.03,0.18) | 0.30(0.17,0.42) | -0.33(-0.67,0.01) | 0.27(0.14,0.39) |
| Sao Tome and Principe | 0.36(0.23,0.49) | 0.48(0.43,0.53) | -0.16(-0.46,0.14) | -0.09(-0.37,0.20) |
| Saudi Arabia | -0.76(-0.93,-0.60) | 0.56(0.49,0.64) | -0.06(-0.55,0.43) | 0.31(0.15,0.47) |
| Senegal | 0.51(0.30,0.72) | 0.46(0.41,0.51) | -0.21(-0.37,-0.04) | -0.14(-0.29,0.02) |
| Serbia | 0.60(0.52,0.69) | 0.27(0.19,0.35) | -1.11(-1.28,-0.95) | -0.39(-0.44,-0.33) |
| Seychelles | 0.42(0.37,0.47) | 0.16(0.13,0.19) | -1.33(-1.54,-1.11) | -0.88(-1.03,-0.72) |
| Sierra Leone | -0.13(-0.26,0.00) | 0.50(0.44,0.57) | 1.38(1.27,1.49) | 1.35(1.25,1.46) |
| Singapore | 0.88(0.63,1.14) | 0.26(0.09,0.42) | -6.13(-6.52,-5.74) | -1.66(-1.83,-1.48) |
| Slovakia | 1.02(0.89,1.15) | 0.20(0.17,0.23) | -0.46(-0.67,-0.25) | -0.08(-0.16,0.00) |
| Slovenia | 0.72(0.51,0.93) | 0.23(-0.02,0.49) | -2.77(-3.28,-2.25) | -0.54(-0.80,-0.27) |
| Solomon Islands | 0.50(0.47,0.53) | -0.12(-0.16,-0.08) | -1.71(-1.87,-1.54) | -1.57(-1.73,-1.42) |
| Somalia | 0.31(0.27,0.34) | -0.14(-0.23,-0.04) | -0.64(-0.79,-0.48) | -0.51(-0.65,-0.37) |
| South Africa | 0.17(0.08,0.27) | 0.38(0.35,0.40) | -0.45(-1.22,0.32) | -0.36(-0.99,0.28) |
| South Sudan | 0.47(0.41,0.53) | -0.17(-0.23,-0.11) | -0.20(-0.34,-0.05) | -0.19(-0.31,-0.06) |
| Spain | 0.18(-0.52,0.87) | -0.42(-1.10,0.26) | -1.54(-1.98,-1.11) | -0.59(-1.23,0.04) |
| Sri Lanka | 1.33(0.98,1.67) | 0.84(0.49,1.19) | -3.42(-3.77,-3.08) | -0.33(-0.54,-0.11) |
| Sudan | 0.98(0.91,1.04) | 1.12(1.00,1.23) | 1.17(0.97,1.37) | 1.10(0.96,1.24) |
| Suriname | 0.52(0.48,0.57) | 0.13(0.09,0.17) | -1.63(-1.89,-1.37) | -1.28(-1.50,-1.06) |
| Sweden | 0.08(-0.05,0.20) | -0.07(-0.28,0.14) | -6.02(-7.14,-4.89) | -0.71(-0.94,-0.48) |
| Switzerland | 0.18(0.13,0.23) | -0.04(-0.12,0.04) | -1.18(-1.69,-0.67) | -0.23(-0.35,-0.11) |
| Syrian Arab Republic | 0.82(0.62,1.02) | 1.03(0.90,1.16) | -1.03(-1.24,-0.81) | 0.01(-0.05,0.08) |
| Taiwan (Province of China) | 2.11(1.34,2.88) | 1.65(0.65,2.65) | -2.97(-3.67,-2.26) | -2.01(-2.74,-1.27) |
| Tajikistan | 0.23(0.14,0.32) | 0.16(0.09,0.23) | -0.35(-0.60,-0.10) | -0.14(-0.32,0.04) |
| Thailand | 0.63(0.58,0.69) | 0.45(0.38,0.52) | 0.92(0.69,1.16) | 0.72(0.59,0.84) |
| Timor-Leste | 0.91(0.84,0.99) | 0.42(0.38,0.46) | -0.76(-1.19,-0.32) | -0.49(-0.85,-0.12) |
| Togo | 0.37(0.28,0.47) | 0.25(0.20,0.30) | 0.37(0.28,0.46) | 0.39(0.31,0.48) |
| Tokelau | 0.20(0.12,0.27) | 0.38(0.33,0.43) | -2.07(-2.24,-1.90) | -1.88(-2.04,-1.72) |
| Tonga | 0.66(0.62,0.70) | 0.06(0.03,0.09) | -1.01(-1.17,-0.85) | -0.94(-1.10,-0.77) |
| Trinidad and Tobago | 0.20(0.06,0.34) | 0.38(0.30,0.45) | -2.20(-2.56,-1.85) | -1.36(-1.58,-1.14) |
| Tunisia | 0.62(0.57,0.66) | 0.89(0.81,0.97) | -0.01(-0.14,0.12) | 0.64(0.56,0.72) |
| Turkmenistan | 0.55(0.47,0.63) | 0.19(0.15,0.23) | -0.45(-0.69,-0.21) | -0.22(-0.37,-0.07) |
| Tuvalu | 0.86(0.80,0.91) | -0.15(-0.19,-0.12) | -1.42(-1.57,-1.26) | -1.36(-1.51,-1.21) |
| T眉rkiye | 0.22(-0.09,0.53) | 1.04(0.58,1.49) | -1.95(-2.03,-1.87) | -0.71(-0.87,-0.55) |
| Uganda | 0.74(0.65,0.82) | 0.60(0.55,0.64) | 0.12(-0.07,0.32) | 0.26(0.12,0.41) |
| Ukraine | 0.86(0.70,1.02) | -0.21(-0.28,-0.14) | -0.73(-1.19,-0.25) | -0.60(-0.93,-0.26) |
| United Arab Emirates | 0.95(0.79,1.11) | 0.68(0.57,0.79) | -1.23(-1.50,-0.97) | 0.11(0.01,0.21) |
| United Kingdom | -0.04(-0.26,0.17) | -0.42(-0.64,-0.20) | 0.44(-0.15,1.03) | -0.20(-0.42,0.02) |
| United Republic of Tanzania | 0.59(0.47,0.71) | 0.41(0.34,0.48) | 0.49(0.27,0.71) | 0.49(0.31,0.67) |
| United States of America | 0.35(0.19,0.50) | -0.20(-0.40,-0.01) | 1.64(1.41,1.86) | 0.24(0.06,0.42) |
| United States Virgin Islands | 0.58(0.52,0.64) | 0.28(0.27,0.30) | -1.31(-1.69,-0.92) | -0.71(-0.97,-0.44) |
| Uruguay | 0.76(0.72,0.79) | -0.01(-0.05,0.04) | -0.99(-1.17,-0.81) | -0.49(-0.55,-0.42) |
| Uzbekistan | 0.76(0.72,0.80) | 0.14(0.08,0.20) | 0.12(-0.53,0.77) | 0.11(-0.18,0.40) |
| Vanuatu | 0.51(0.49,0.54) | -0.06(-0.10,-0.03) | -1.46(-1.63,-1.29) | -1.39(-1.55,-1.23) |
| Venezuela (Bolivarian Republic of) | -0.15(-0.36,0.07) | -0.18(-0.36,-0.00) | 0.48(0.14,0.82) | 0.33(0.04,0.63) |
| Viet Nam | 0.81(0.74,0.87) | 0.36(0.33,0.39) | -1.58(-1.77,-1.39) | -0.92(-1.05,-0.80) |
| Yemen | 0.83(0.79,0.86) | 1.00(0.88,1.13) | 1.15(0.82,1.48) | 1.07(0.89,1.25) |
| Zambia | 0.87(0.76,0.98) | 0.40(0.30,0.49) | 0.25(0.10,0.40) | 0.29(0.18,0.40) |

**Supplementary Figure1**

Results of the age-period-cohort analysis of incidence (A. Net drifts and local drifts of incidence; B. Age effects on incidence; C. Period effects on incidence; D. Cohort effects on incidence)


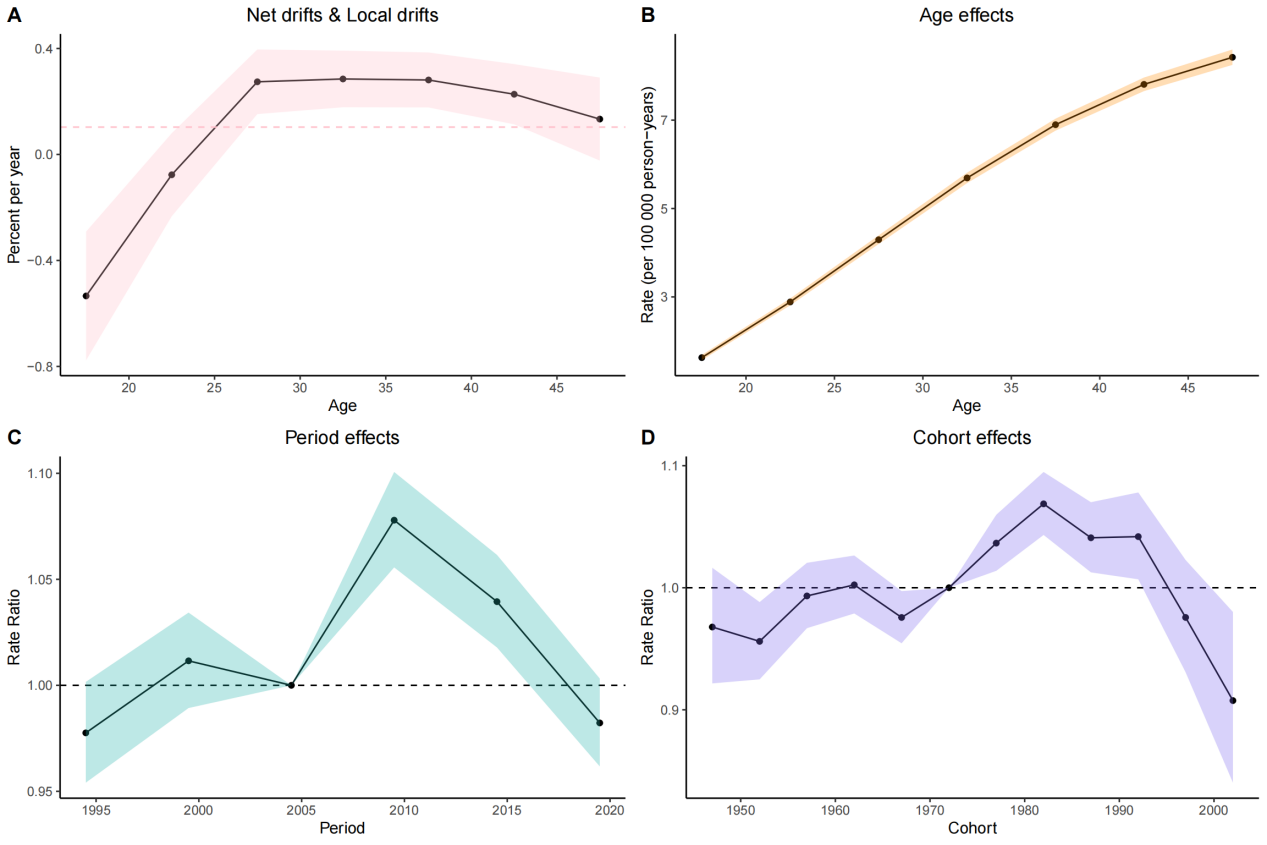


**Supplementary Figure2**

Results of the age-period-cohort analysis of prevalence (A. Net drifts and local drifts of prevalence; B. Age effects on prevalence; C. Period effects on prevalence; D. Cohort effects on prevalence)


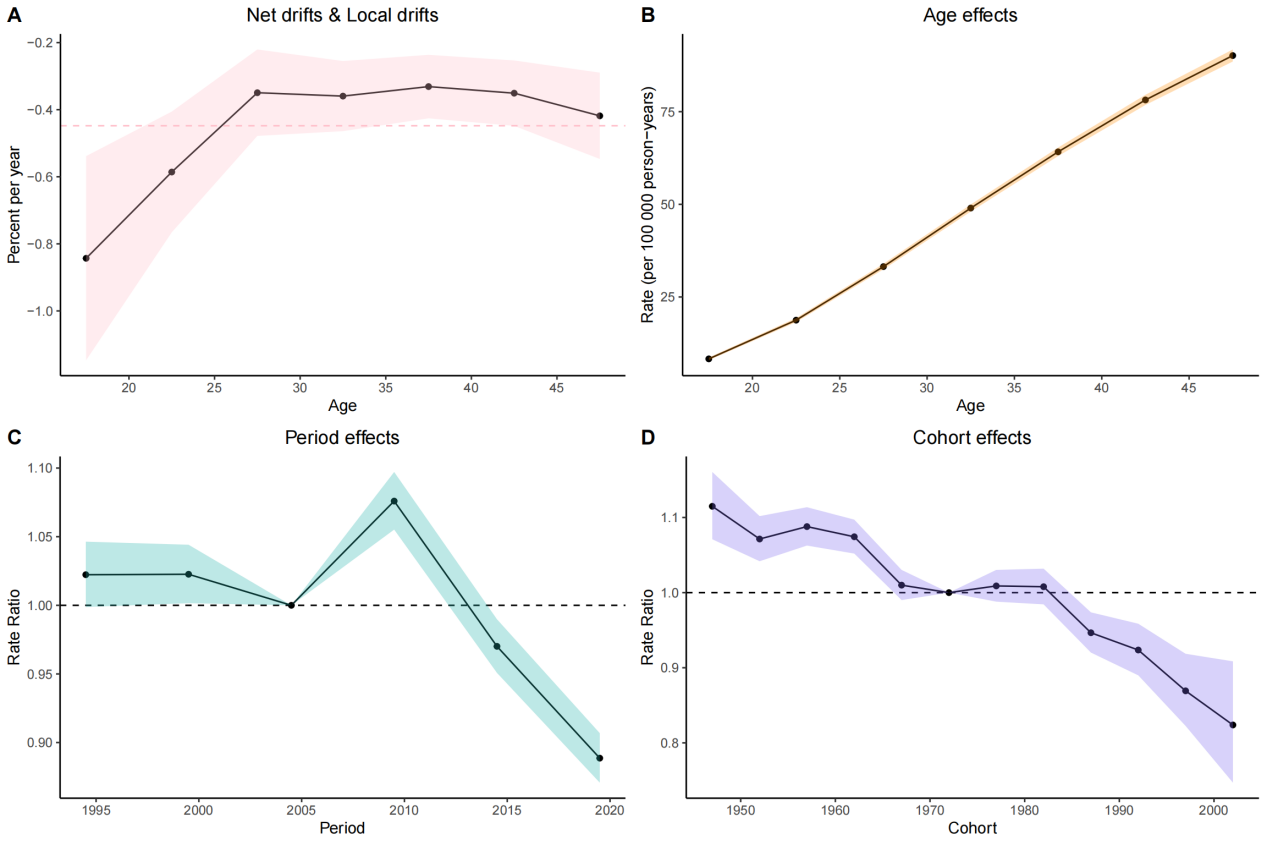


**Supplementary Figure3**

Results of the age-period-cohort analysis of mortality (A. Net drifts and local drifts of mortality; B. Age effects on mortality; C. Period effects on mortality; D. Cohort effects on mortality)


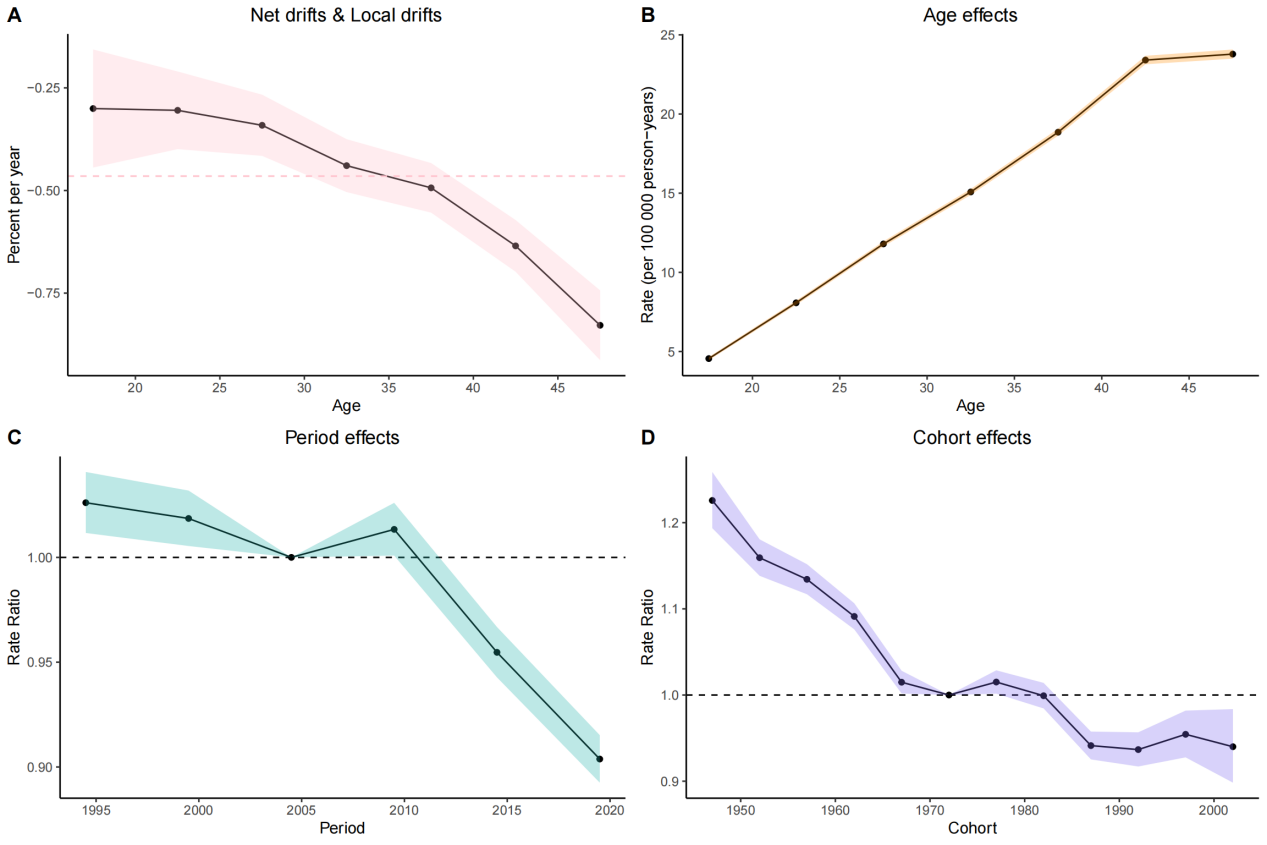


**Supplementary Figure4**

Results of the age-period-cohort analysis of DALYs (A. Net drifts and local drifts of DALYs; B. Age effects on DALYs; C. Period effects on DALYs; D. Cohort effects on DALYs)


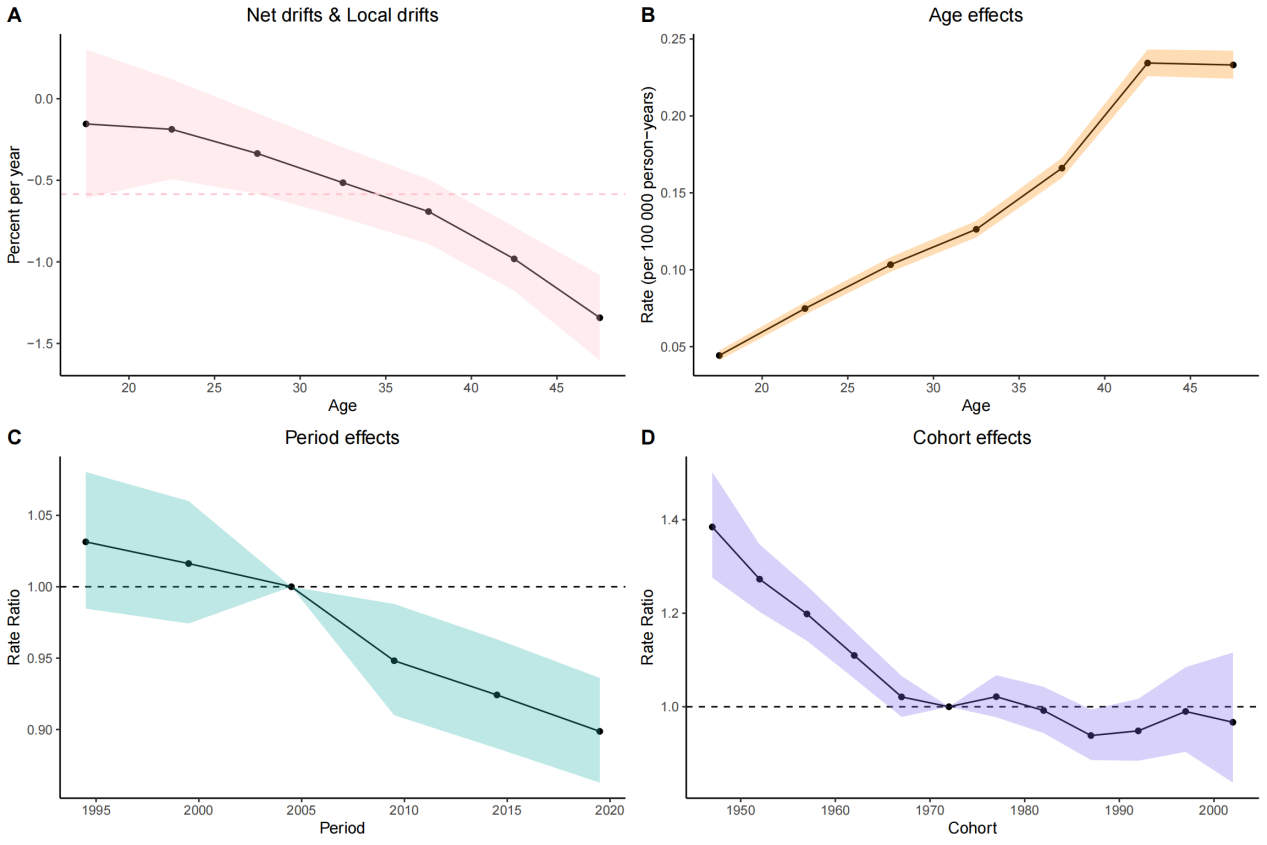


**Supplementary Figure5**

Global trends in age-standardized rates of IBD from 1990 to 2021, stratified by sex (A. Incidence; B. Prevalence; C. Mortality; D. DALYs).


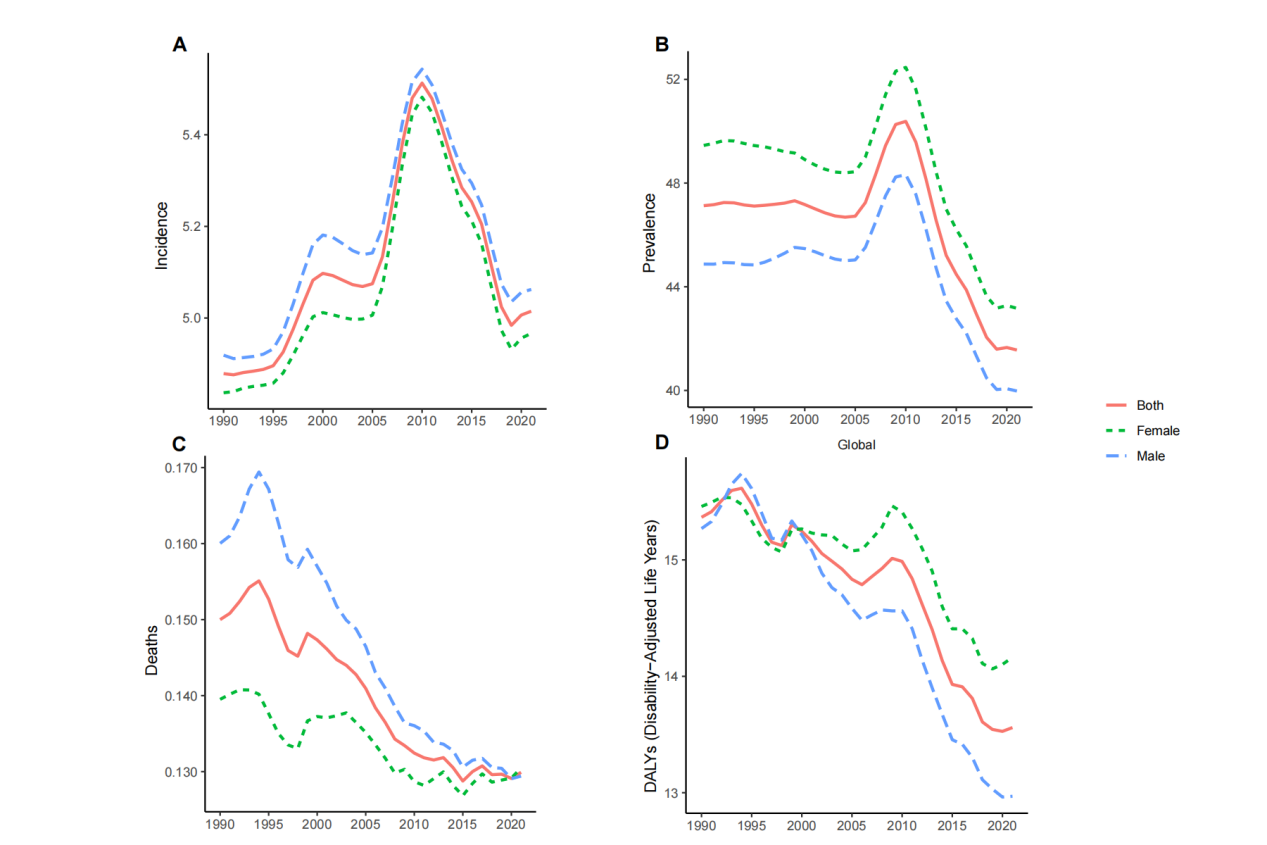


**Supplementary Figure6**

Global temporal trends in age-specific rates of IBD from 1990 to 2021 (A. Incidence; B. Prevalence; C. Mortality; D. DALYs).


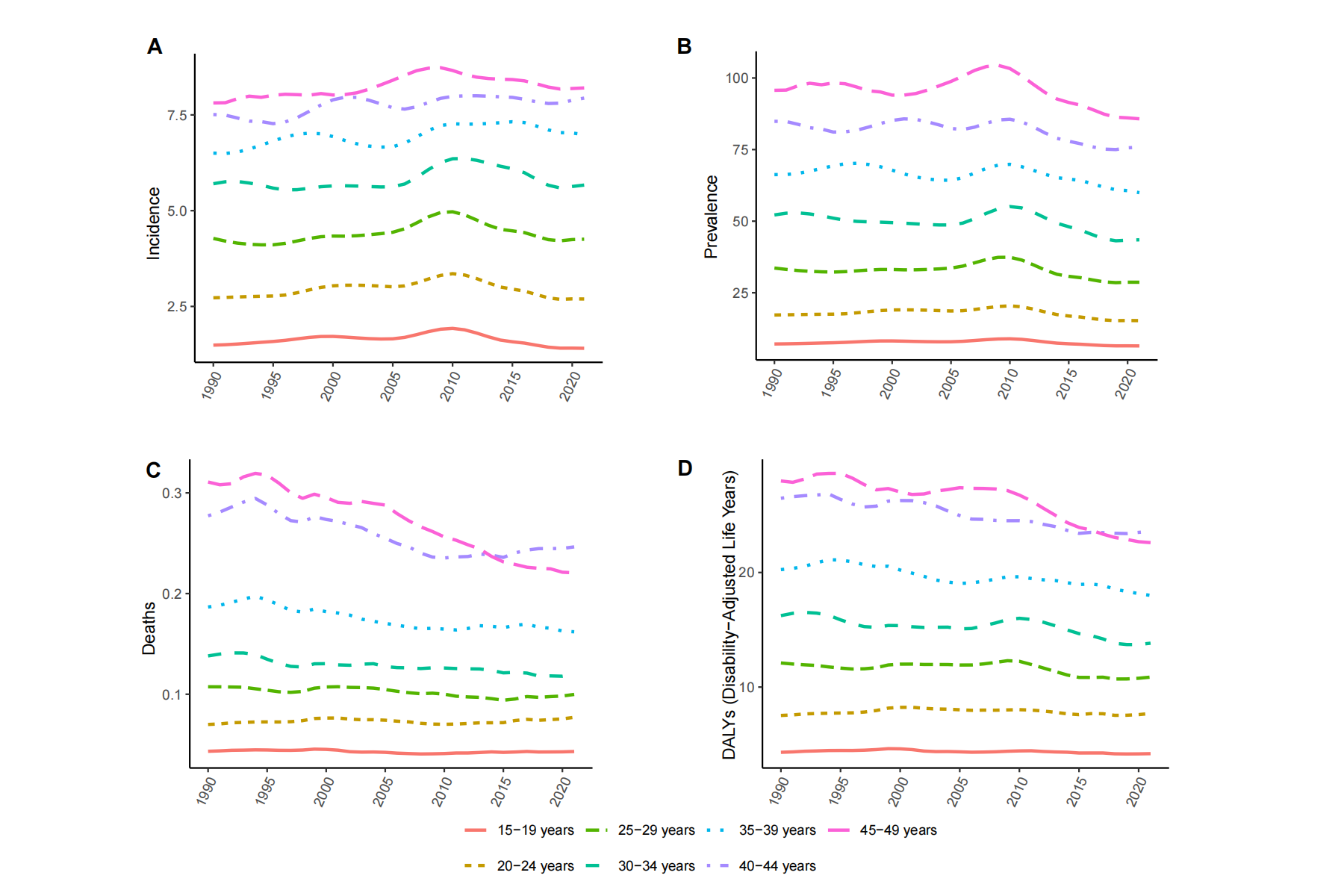

Supplement: Supplementary file 1 — Table S1: EAPCs of age‐standardized rates (incidence, prevalence, mortality, and DALYs) in 204 countries worldwide. Figure S1: Results of the age‐period‐cohort analysis of incidence (A. Net drifts and local drifts of incidence; B. Age effects on incidence; C. Period effects on incidence; D. Cohort effects on incidence). Figure S2: Results of the age‐period‐cohort analysis of prevalence (A. Net drifts and local drifts of prevalence; B. Age effects on prevalence; C. Period effects on prevalence; D. Cohort effects on prevalence). Figure S3: Results of the age‐period‐cohort analysis of mortality (A. Net drifts and local drifts of mortality; B. Age effects on mortality; C. Period effects on mortality; D. Cohort effects on mortality). Figure S4: Results of the age‐period‐cohort analysis of DALYs (A. Net drifts and local drifts of DALYs; B. Age effects on DALYs; C. Period effects on DALYs; D. Cohort effects on DALYs). Figure S5: Global trends in age‐standardized rates of IBD from 1990 to 2021, stratified by sex (A. Incidence; B. Prevalence; C. Mortality; D. DALYs). Figure S6: Global temporal trends in age‐specific rates of IBD from 1990 to 2021 (A. Incidence; B. Prevalence; C. Mortality; D. DALYs). [file JGH3-9-e70282-s001.docx]
