# Supplementary material for: Global Trends and Future Projections in the Burden of Inflammatory Bowel Disease Among Adolescents and Young Adults (15–49 Years) From 1990 to 2021
Source: JGH Open. 2025 Sep 18;9(9):e70282. doi: 10.1002/jgh3.70282 (PMC12446572; doi:10.1002/jgh3.70282)
Supplement: Supplementary file 2 — Data S1: Supporting Information. [file JGH3-9-e70282-s002.zip › supplement material/Global Maps of EAPCs for Disease Burden Indicators (Incidence, Prevalence, Mortality, DALYs/EAPCs of ASIR.pdf]

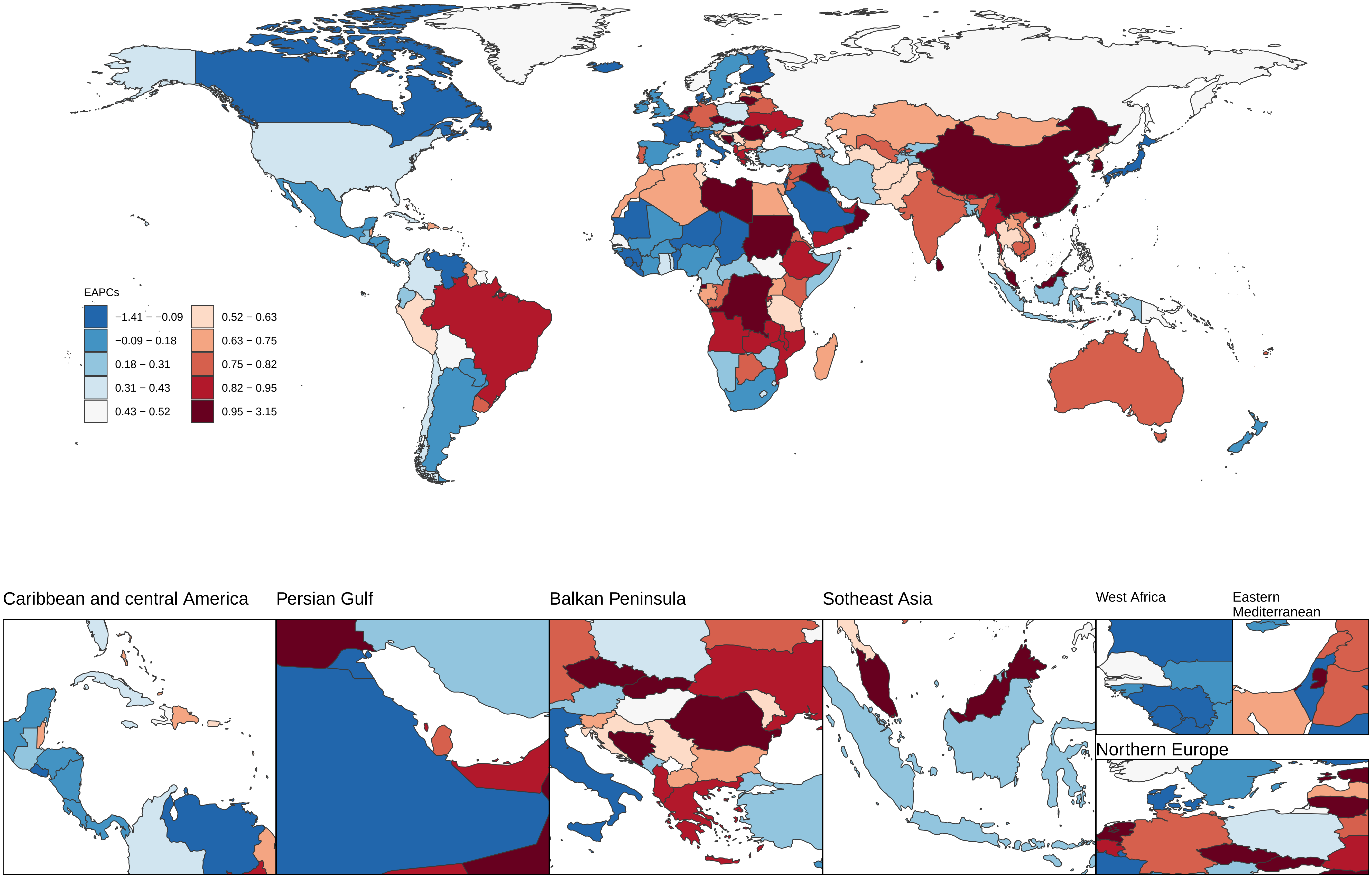
EAPCs
−1.41 − −0.09
−0.09 − 0.18
0.18 − 0.31
0.31 − 0.43
0.43 − 0.52
0.52 − 0.63
0.63 − 0.75
0.75 − 0.82
0.82 − 0.95
0.95 − 3.15
Caribbean and central America
Persian Gulf
Balkan Peninsula
Sotheast Asia
West Africa
Eastern Mediterranean
Northern Europe
