# Supplementary material for: Global Trends and Future Projections in the Burden of Inflammatory Bowel Disease Among Adolescents and Young Adults (15–49 Years) From 1990 to 2021
Source: JGH Open. 2025 Sep 18;9(9):e70282. doi: 10.1002/jgh3.70282 (PMC12446572; doi:10.1002/jgh3.70282)
Supplement: Supplementary file 2 — Data S1: Supporting Information. [file JGH3-9-e70282-s002.zip › supplement material/Global Maps of EAPCs for Disease Burden Indicators (Incidence, Prevalence, Mortality, DALYs/EAPCs of ASPR.pdf]

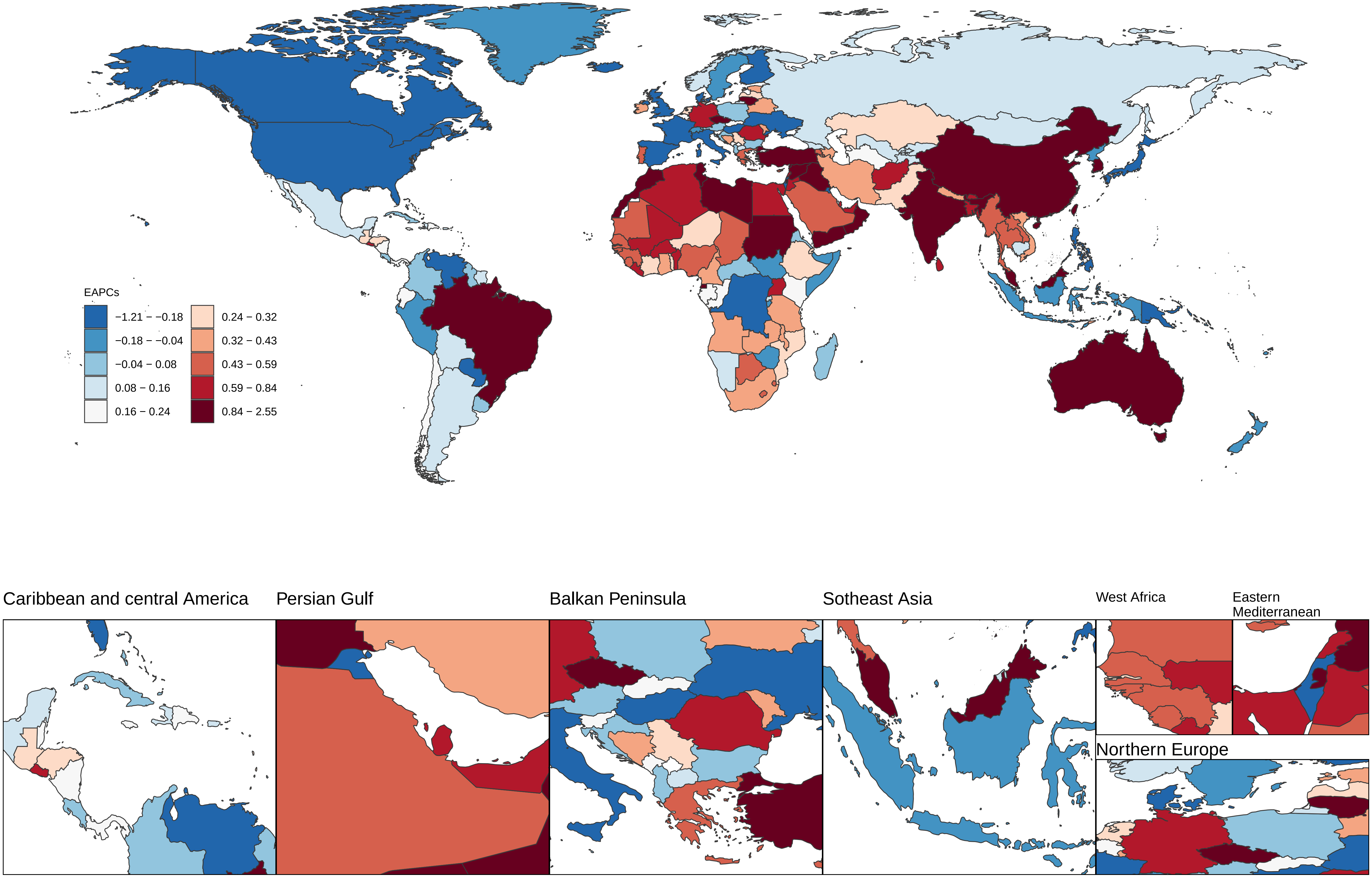
EAPCs
−1.21 − −0.18
−0.18 − −0.04
−0.04 − 0.08
0.08 − 0.16
0.16 − 0.24
0.24 − 0.32
0.32 − 0.43
0.43 − 0.59
0.59 − 0.84
0.84 − 2.55
Caribbean and central America
Persian Gulf
Balkan Peninsula
Sotheast Asia
West Africa
Eastern Mediterranean
Northern Europe
