# Supplementary material for: Global Trends and Future Projections in the Burden of Inflammatory Bowel Disease Among Adolescents and Young Adults (15–49 Years) From 1990 to 2021
Source: JGH Open. 2025 Sep 18;9(9):e70282. doi: 10.1002/jgh3.70282 (PMC12446572; doi:10.1002/jgh3.70282)
Supplement: Supplementary file 2 — Data S1: Supporting Information. [file JGH3-9-e70282-s002.zip › supplement material/Global Maps of EAPCs for Disease Burden Indicators (Incidence, Prevalence, Mortality, DALYs/EAPCs of ASDR.pdf]

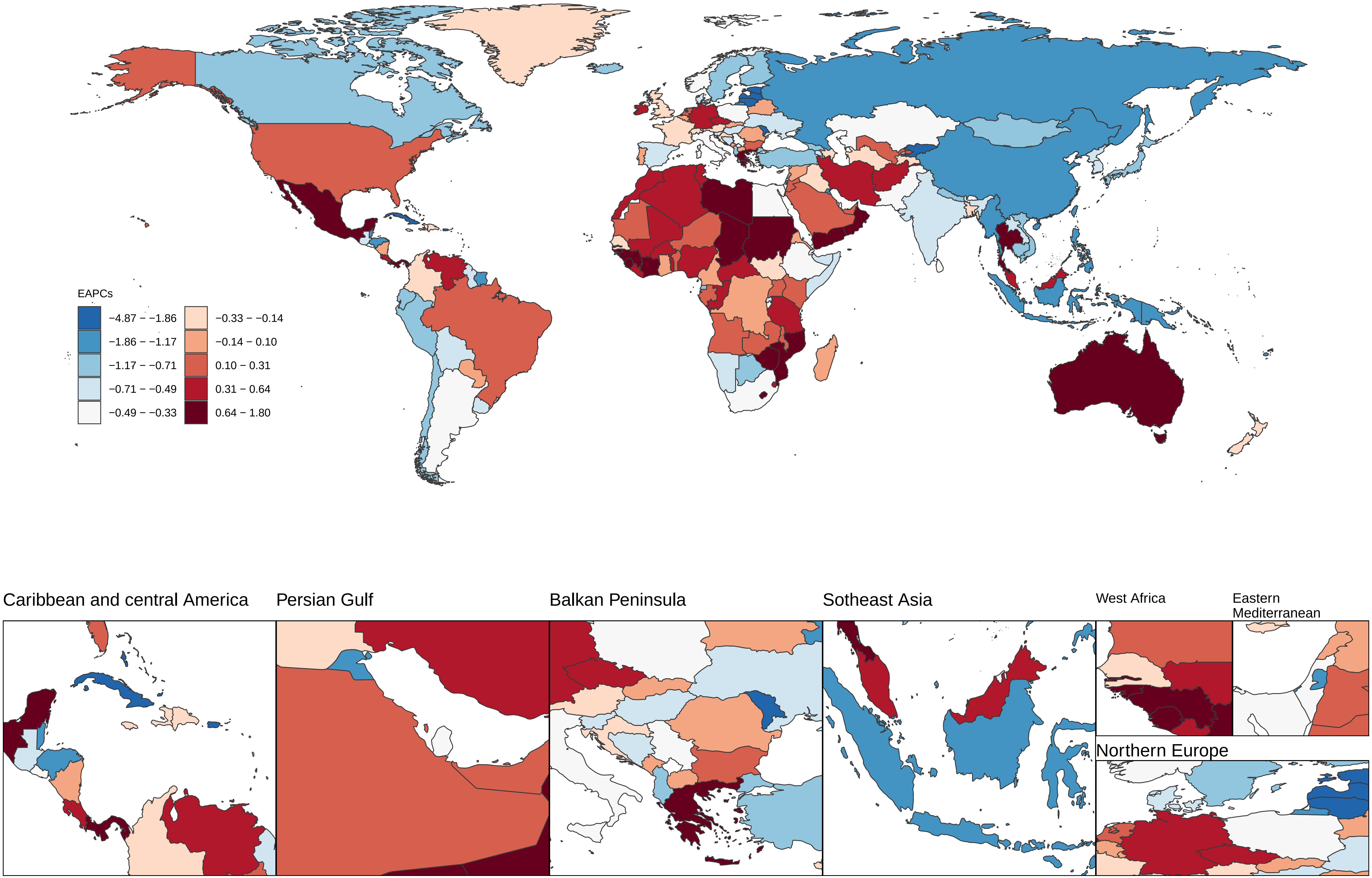
EAPCs
−4.87 − −1.86
−1.86 − −1.17
−1.17 − −0.71
−0.71 − −0.49
−0.49 − −0.33
−0.33 − −0.14
−0.14 − 0.10
0.10 − 0.31
0.31 − 0.64
0.64 − 1.80
Caribbean and central America
Persian Gulf
Balkan Peninsula
Sotheast Asia
West Africa
Eastern Mediterranean
Northern Europe
