# Supplementary material for: Global Trends and Future Projections in the Burden of Inflammatory Bowel Disease Among Adolescents and Young Adults (15–49 Years) From 1990 to 2021
Source: JGH Open. 2025 Sep 18;9(9):e70282. doi: 10.1002/jgh3.70282 (PMC12446572; doi:10.1002/jgh3.70282)
Supplement: Supplementary file 2 — Data S1: Supporting Information. [file JGH3-9-e70282-s002.zip › supplement material/Global Maps of EAPCs for Disease Burden Indicators (Incidence, Prevalence, Mortality, DALYs/EAPCs of ASMR.pdf]

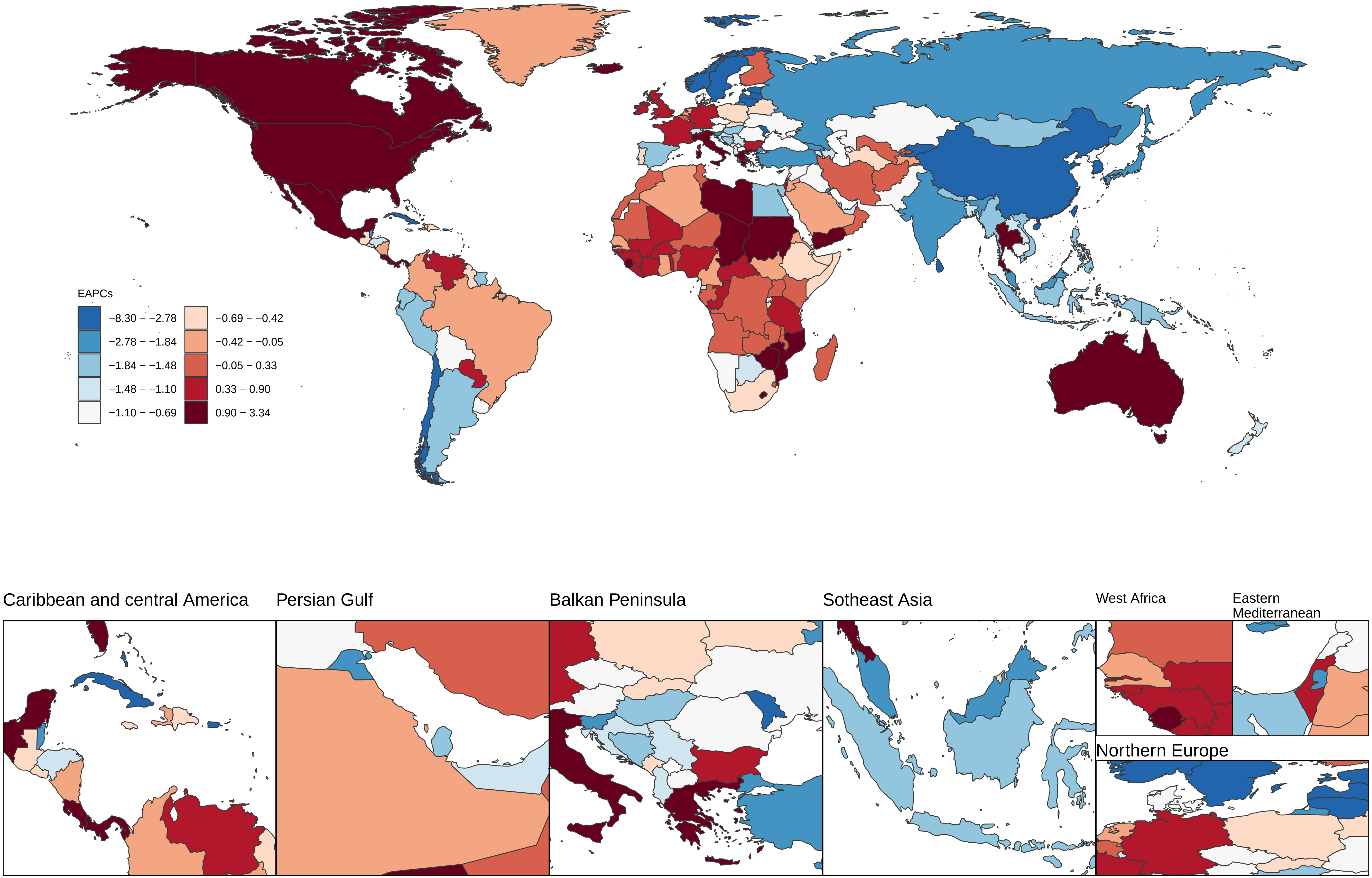
EAPCs
−8.30 − −2.78
−2.78 − −1.84
−1.84 − −1.48
−1.48 − −1.10
−1.10 − −0.69
−0.69 − −0.42
−0.42 − −0.05
−0.05 − 0.33
0.33 − 0.90
0.90 − 3.34
Caribbean and central America
Persian Gulf
Balkan Peninsula
Sotheast Asia
West Africa
Eastern Mediterranean
Northern Europe
