# Supplementary figures and images for: Global Trends and Future Projections in the Burden of Inflammatory Bowel Disease Among Adolescents and Young Adults (15–49 Years) From 1990 to 2021
Source: JGH Open. 2025 Sep 18;9(9):e70282. doi: 10.1002/jgh3.70282 (PMC12446572; doi:10.1002/jgh3.70282)

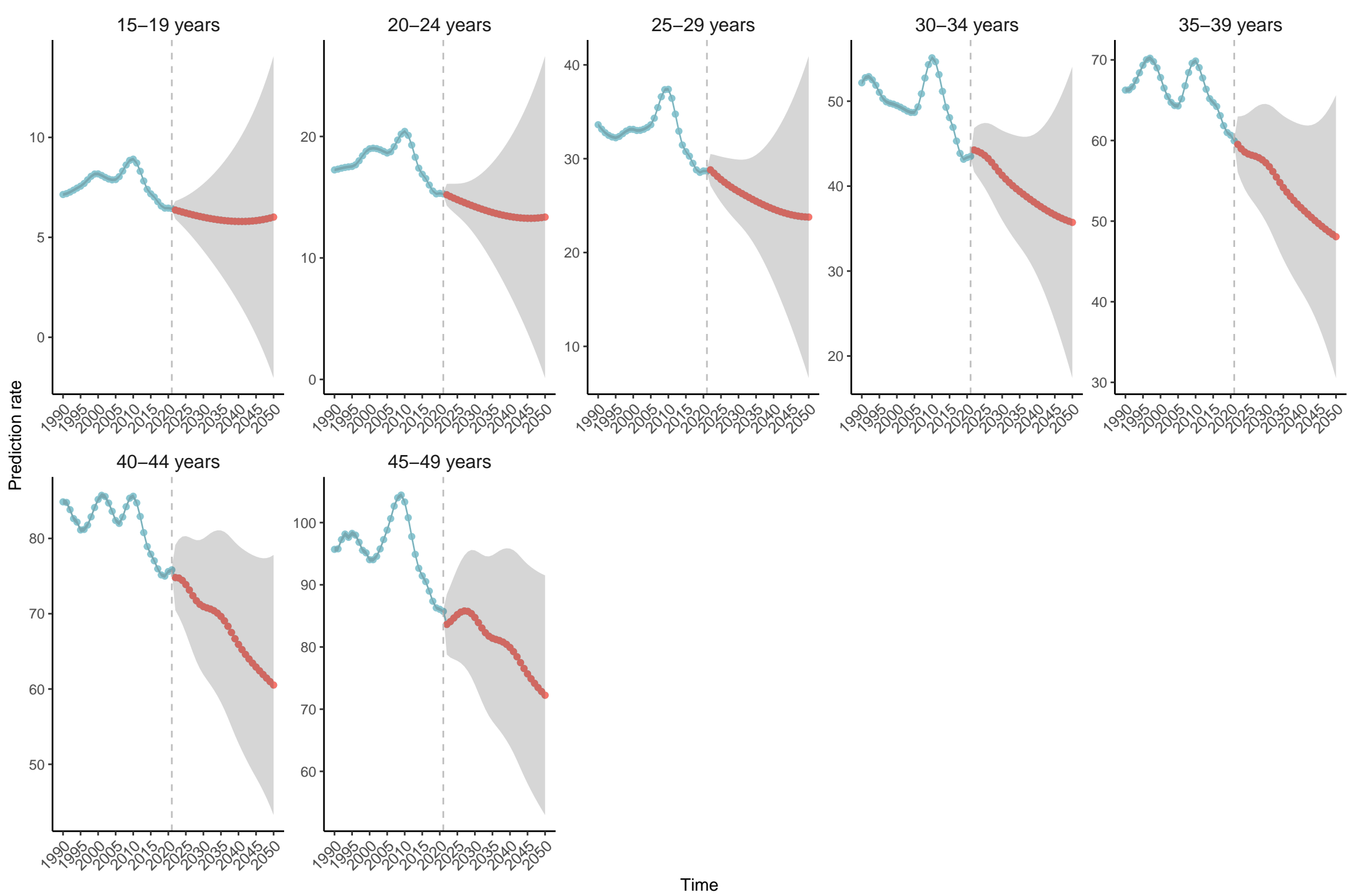

Supplement: Supplementary file 2 — Data S1: Supporting Information. [file JGH3-9-e70282-s002.zip › supplement material/Prediction of Disease Burden for Different Age Group among 15-49/Prediction of ASPR for Different Age Group among 15-49.pdf]

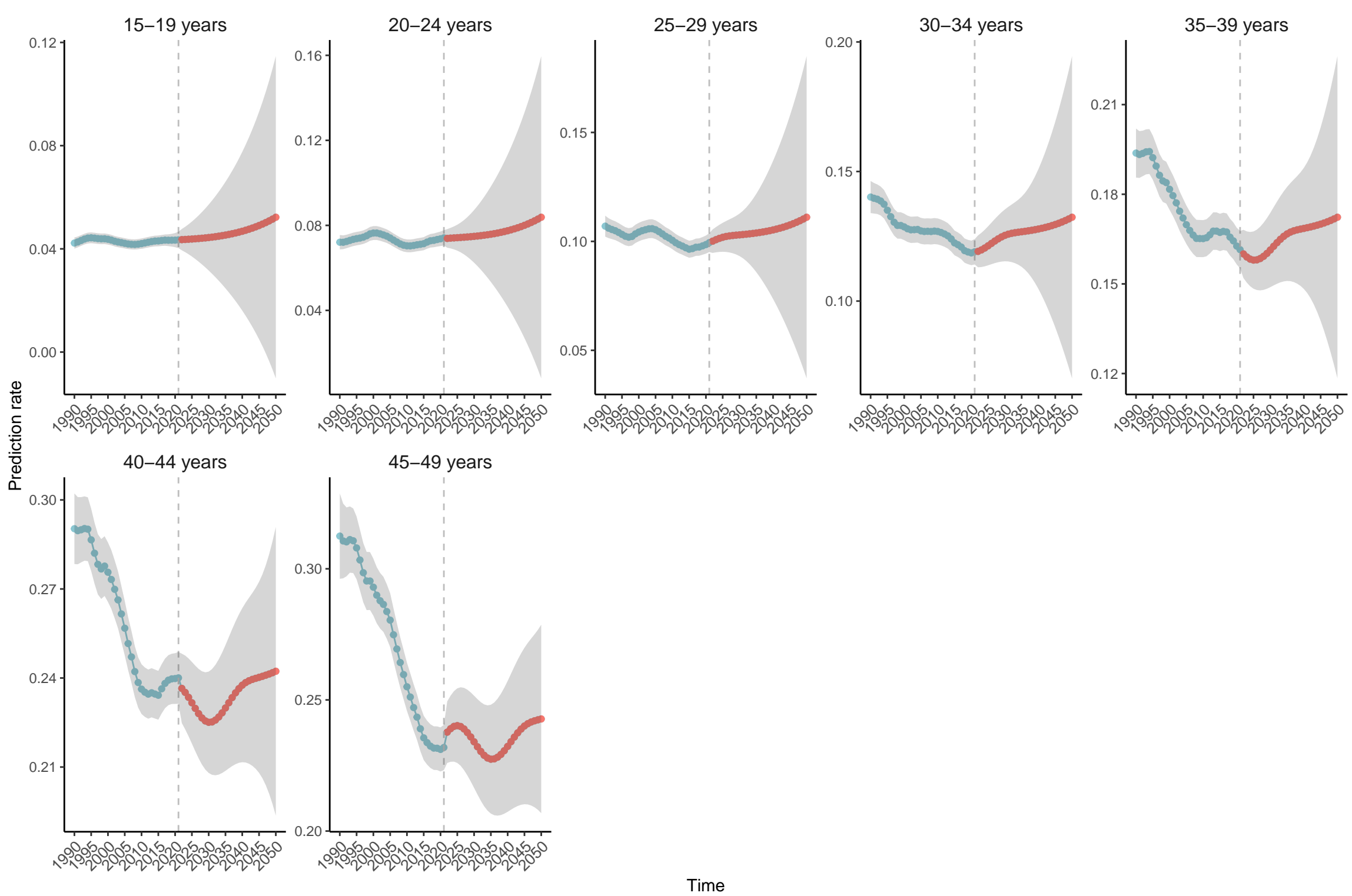

Supplement: Supplementary file 2 — Data S1: Supporting Information. [file JGH3-9-e70282-s002.zip › supplement material/Prediction of Disease Burden for Different Age Group among 15-49/Prediction of ASMR for Different Age Group among 15-49.pdf]

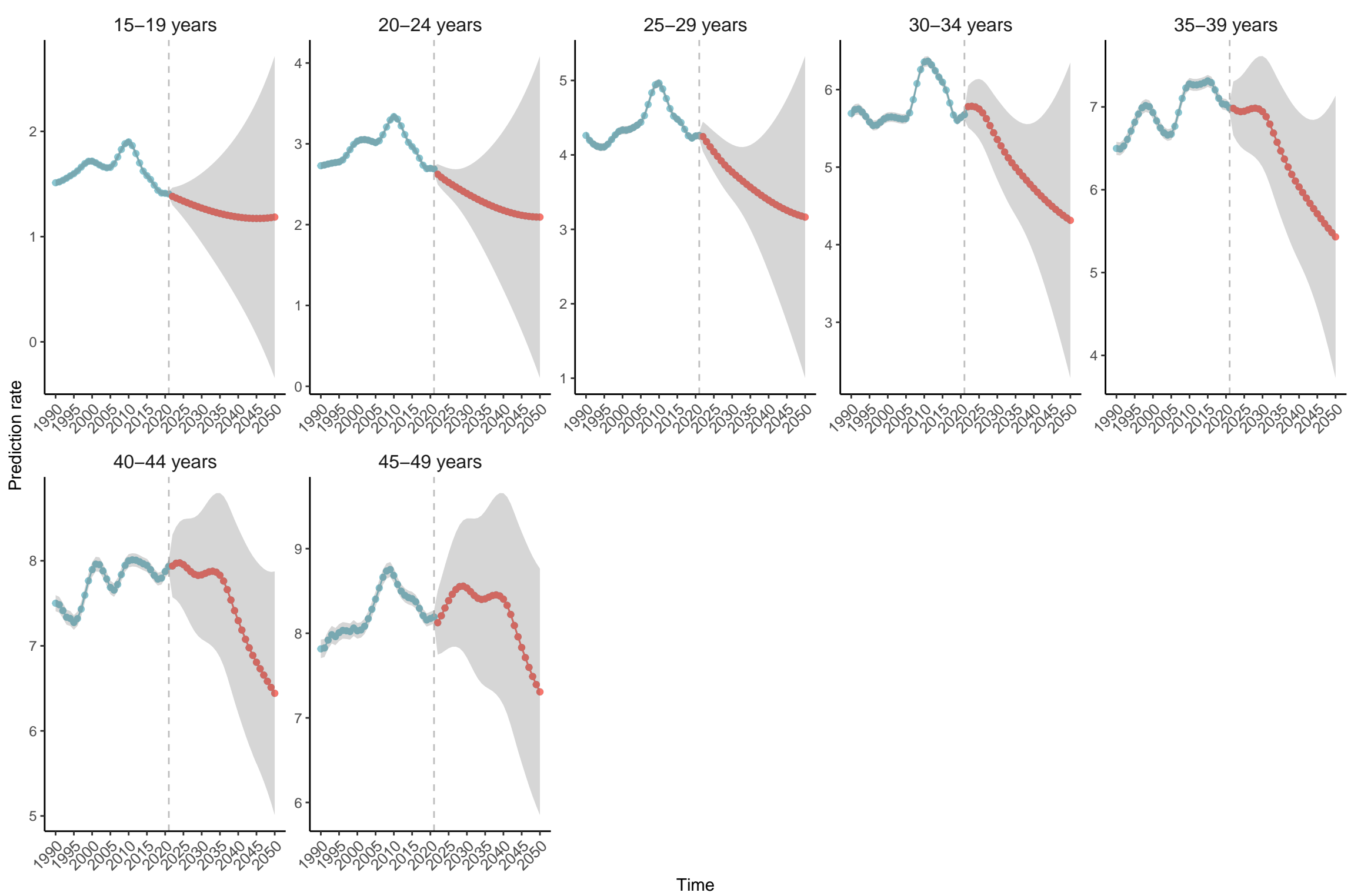

Supplement: Supplementary file 2 — Data S1: Supporting Information. [file JGH3-9-e70282-s002.zip › supplement material/Prediction of Disease Burden for Different Age Group among 15-49/Prediction of ASIR for Different Age Group among 15-49.pdf]

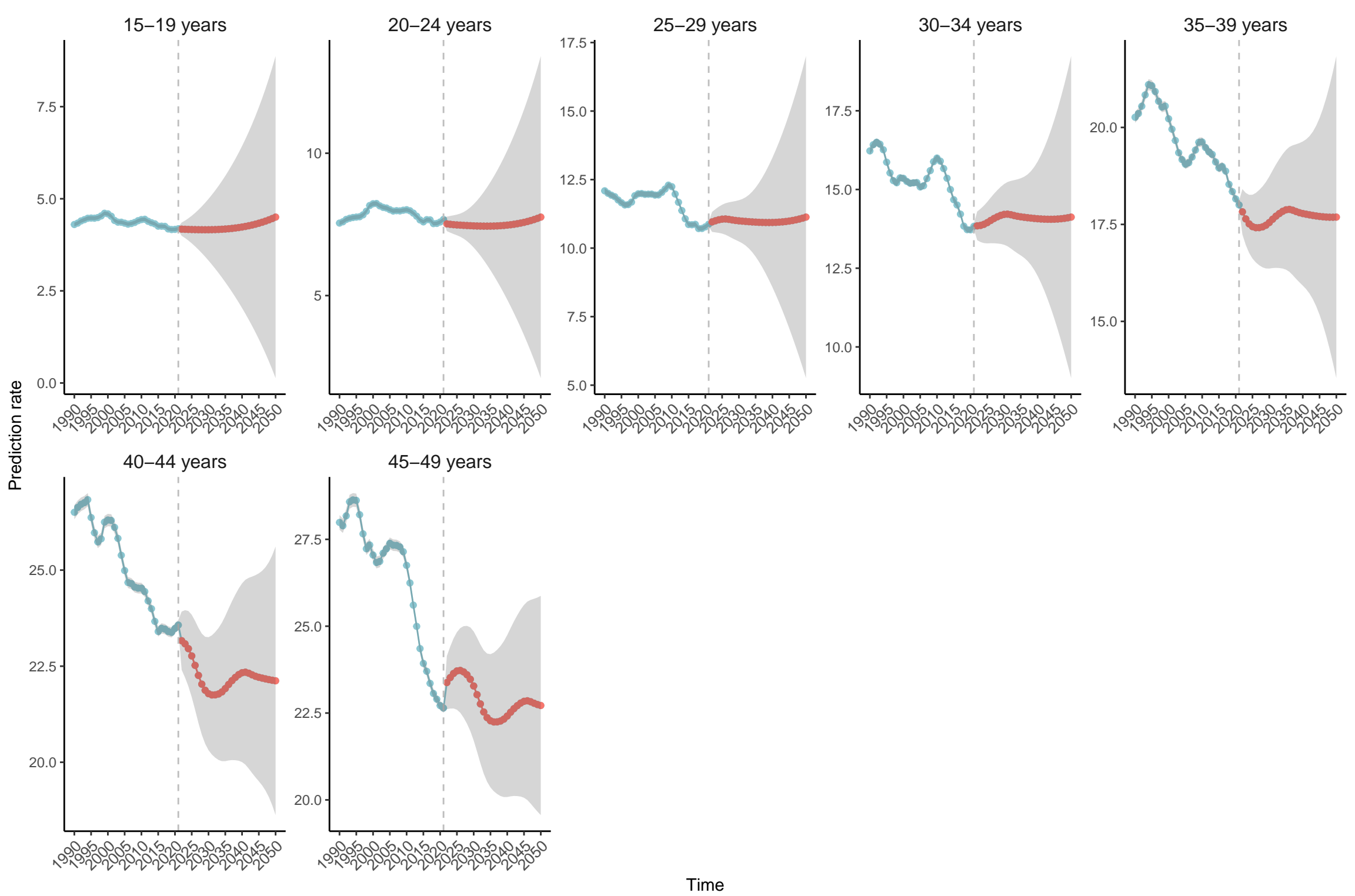

Supplement: Supplementary file 2 — Data S1: Supporting Information. [file JGH3-9-e70282-s002.zip › supplement material/Prediction of Disease Burden for Different Age Group among 15-49/Prediction of ASDR for Different Age Group among 15-49.pdf]

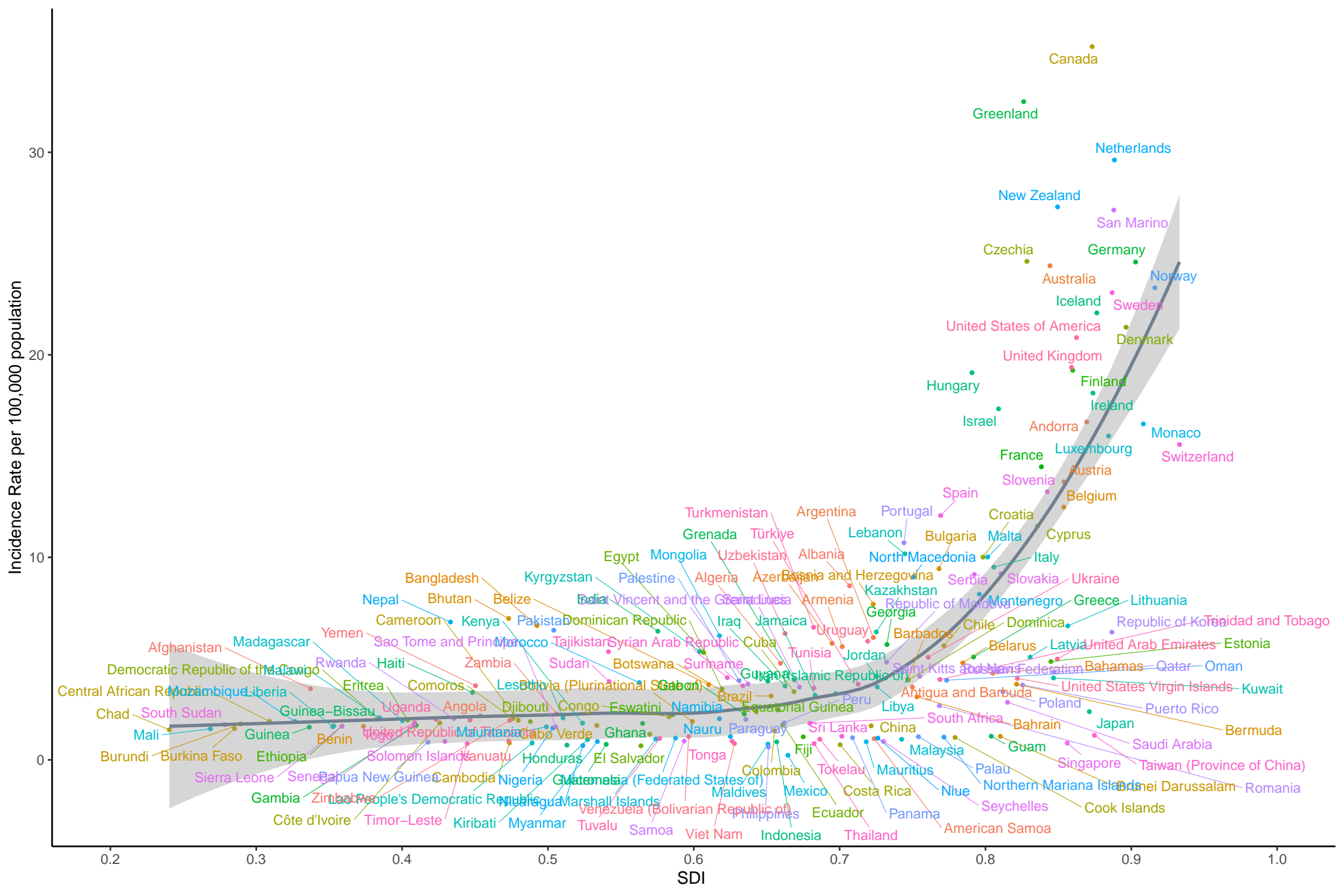

Supplement: Supplementary file 2 — Data S1: Supporting Information. [file JGH3-9-e70282-s002.zip › supplement material/Analysis of the Relationship Between Disease Burden Indicators and SDI/Trends of Age-Standardized Incidence Rates by SDI for 204 Countries and Regions.pdf]

DALYs (Disability-Adjusted Life Years) Rate per 100,000 population

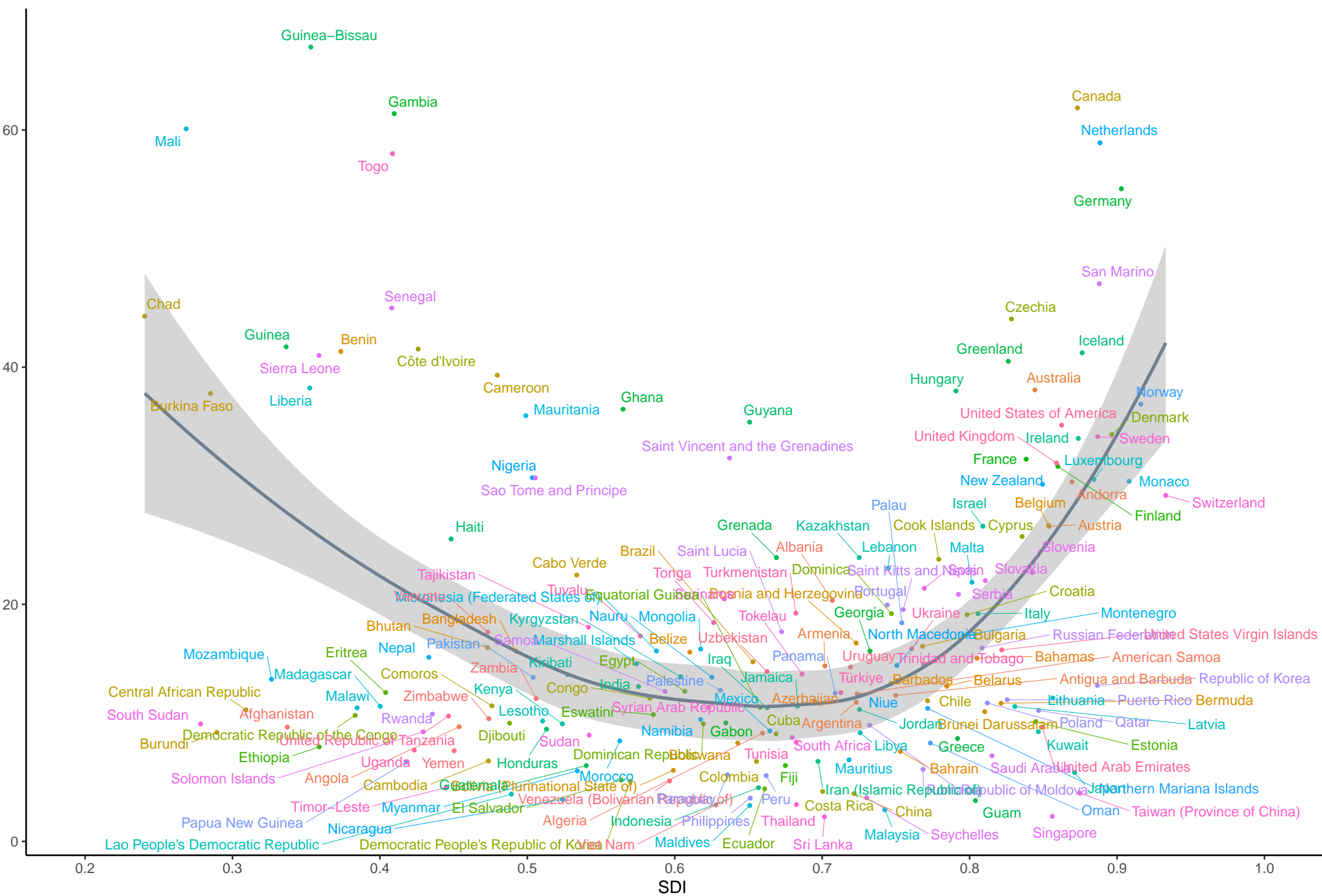

Supplement: Supplementary file 2 — Data S1: Supporting Information. [file JGH3-9-e70282-s002.zip › supplement material/Analysis of the Relationship Between Disease Burden Indicators and SDI/Trends of Age-Standardized DALYs Rates by SDI for 204 Countries and Regions.pdf]

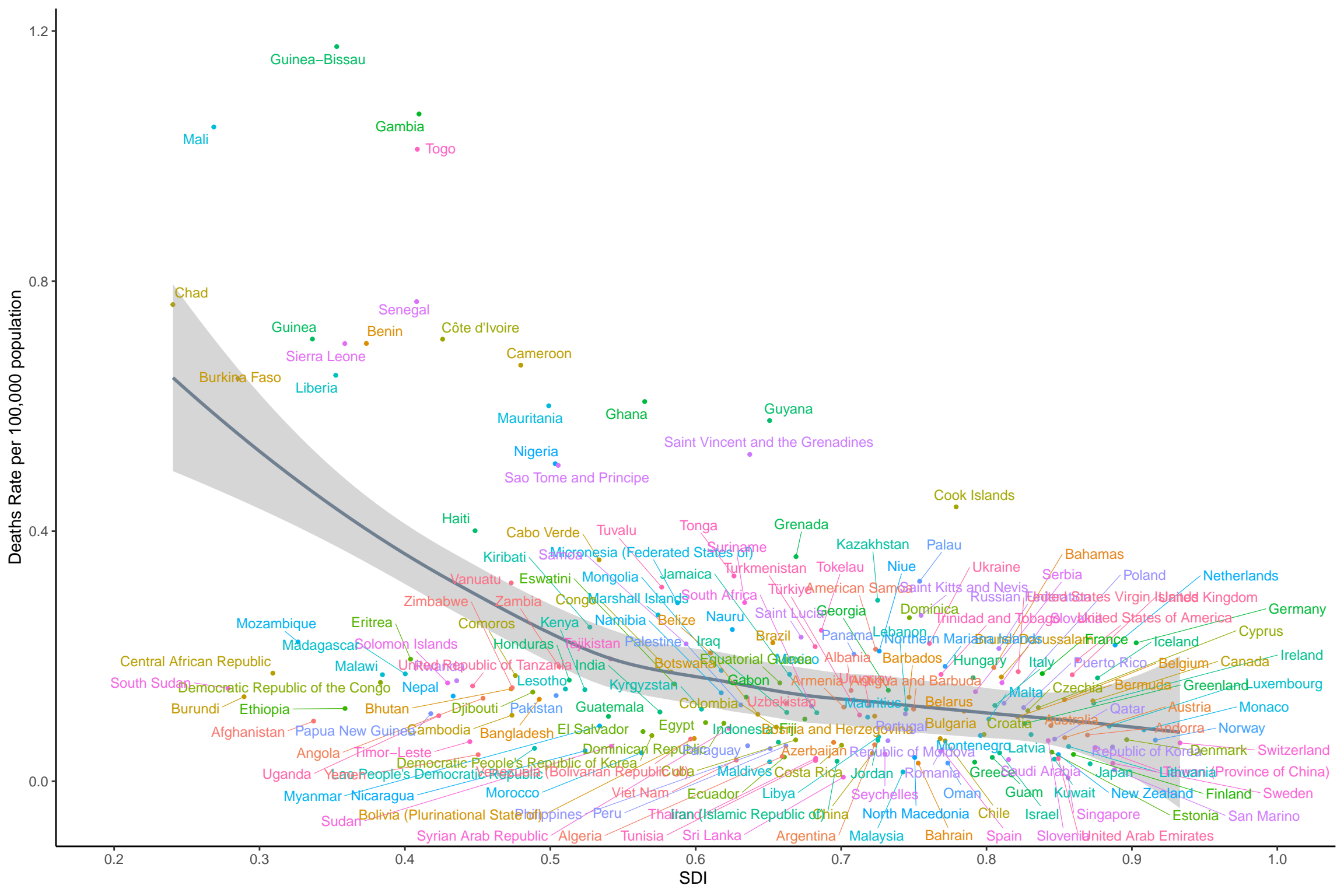

Supplement: Supplementary file 2 — Data S1: Supporting Information. [file JGH3-9-e70282-s002.zip › supplement material/Analysis of the Relationship Between Disease Burden Indicators and SDI/Trends of Age-Standardized Mortality Rates by SDI for 204 Countries and Regions.pdf]

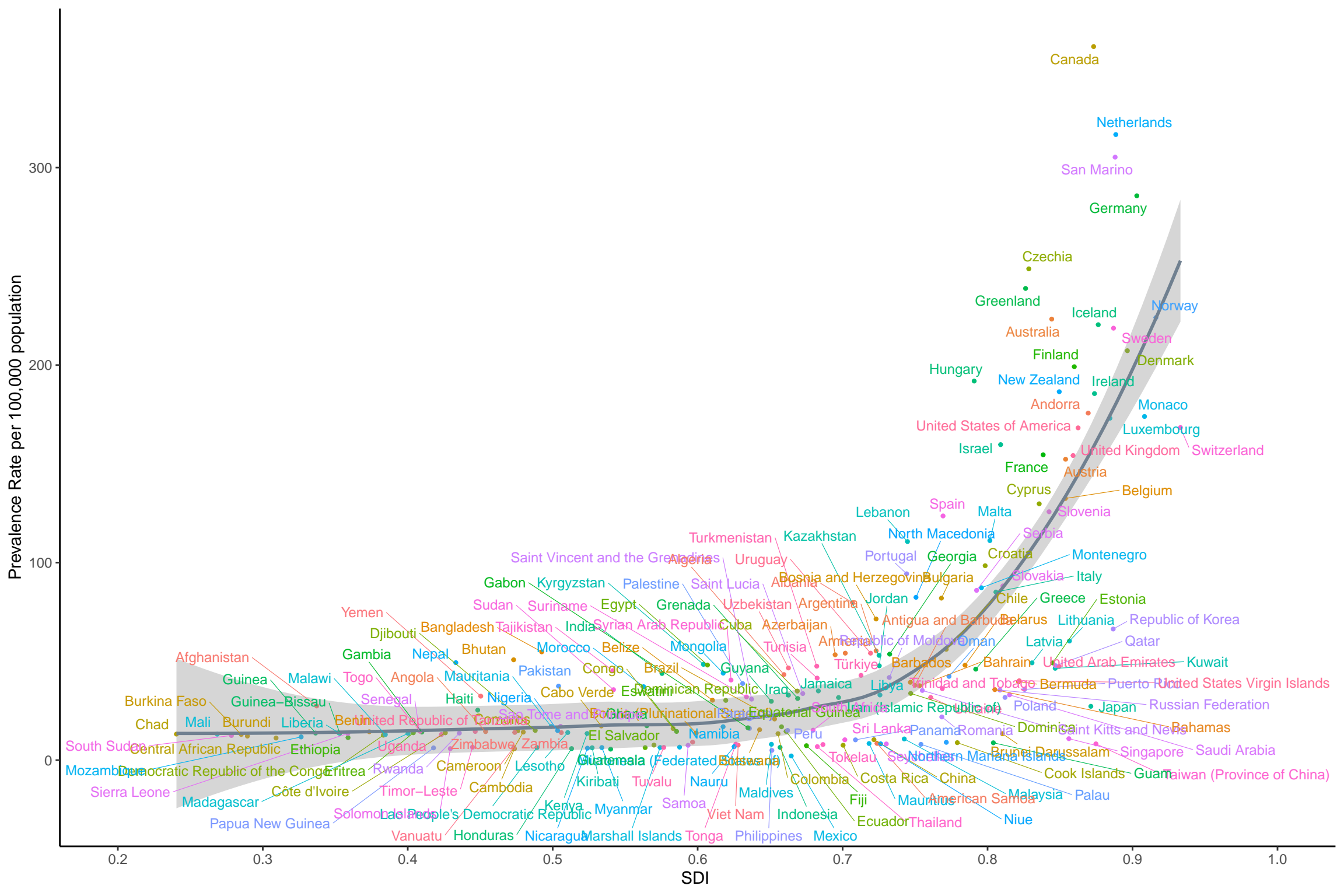

Supplement: Supplementary file 2 — Data S1: Supporting Information. [file JGH3-9-e70282-s002.zip › supplement material/Analysis of the Relationship Between Disease Burden Indicators and SDI/Trends of Age-Standardized Prevalence Rates by SDI for 204 Countries and Regions.pdf]

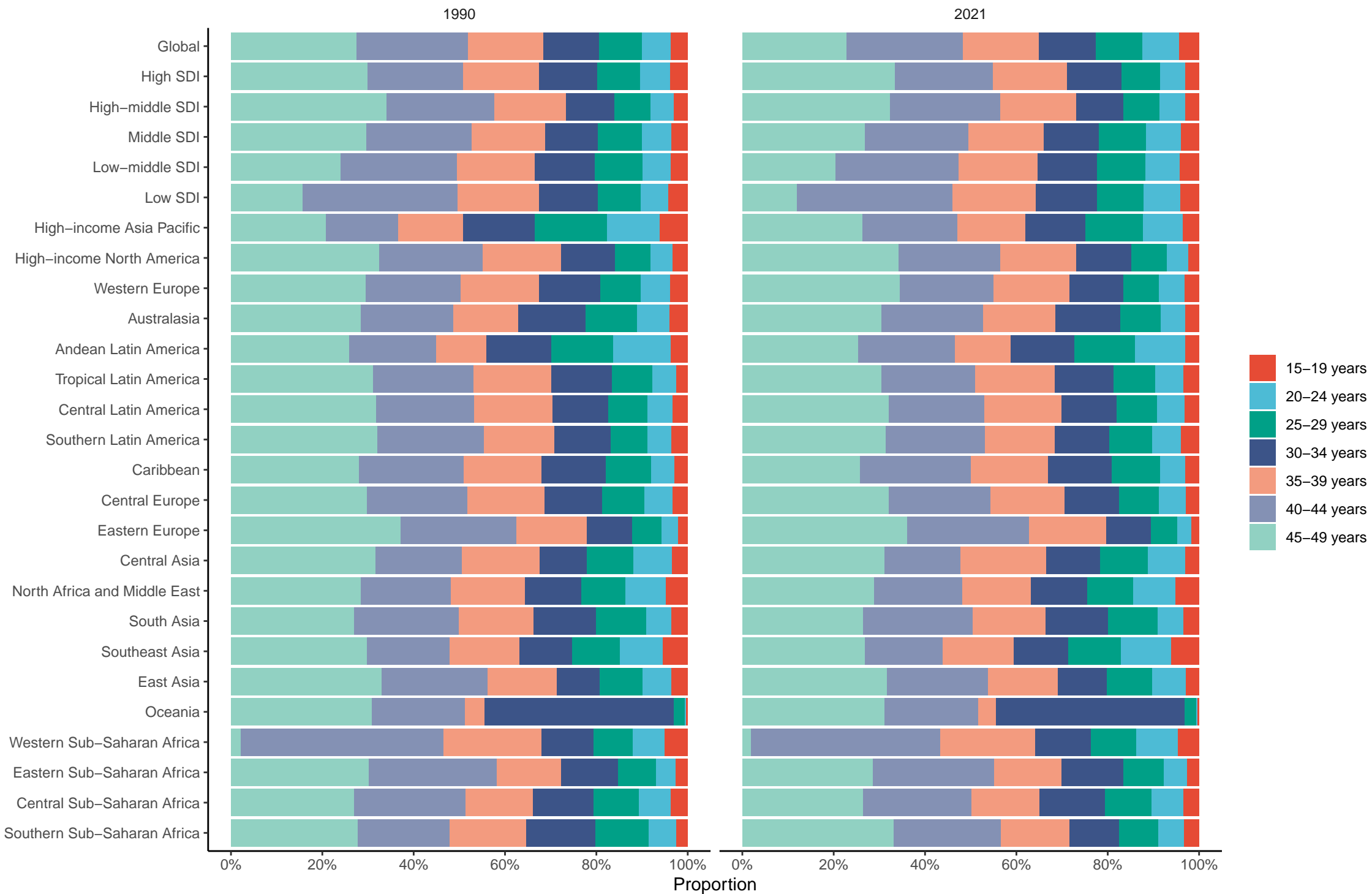

Supplement: Supplementary file 2 — Data S1: Supporting Information. [file JGH3-9-e70282-s002.zip › supplement material/Proportions of Age-Standardized Rates by Age Group/death/Different Regions Proportions of Age-Standardized Mortality Rates by Age Group.pdf]

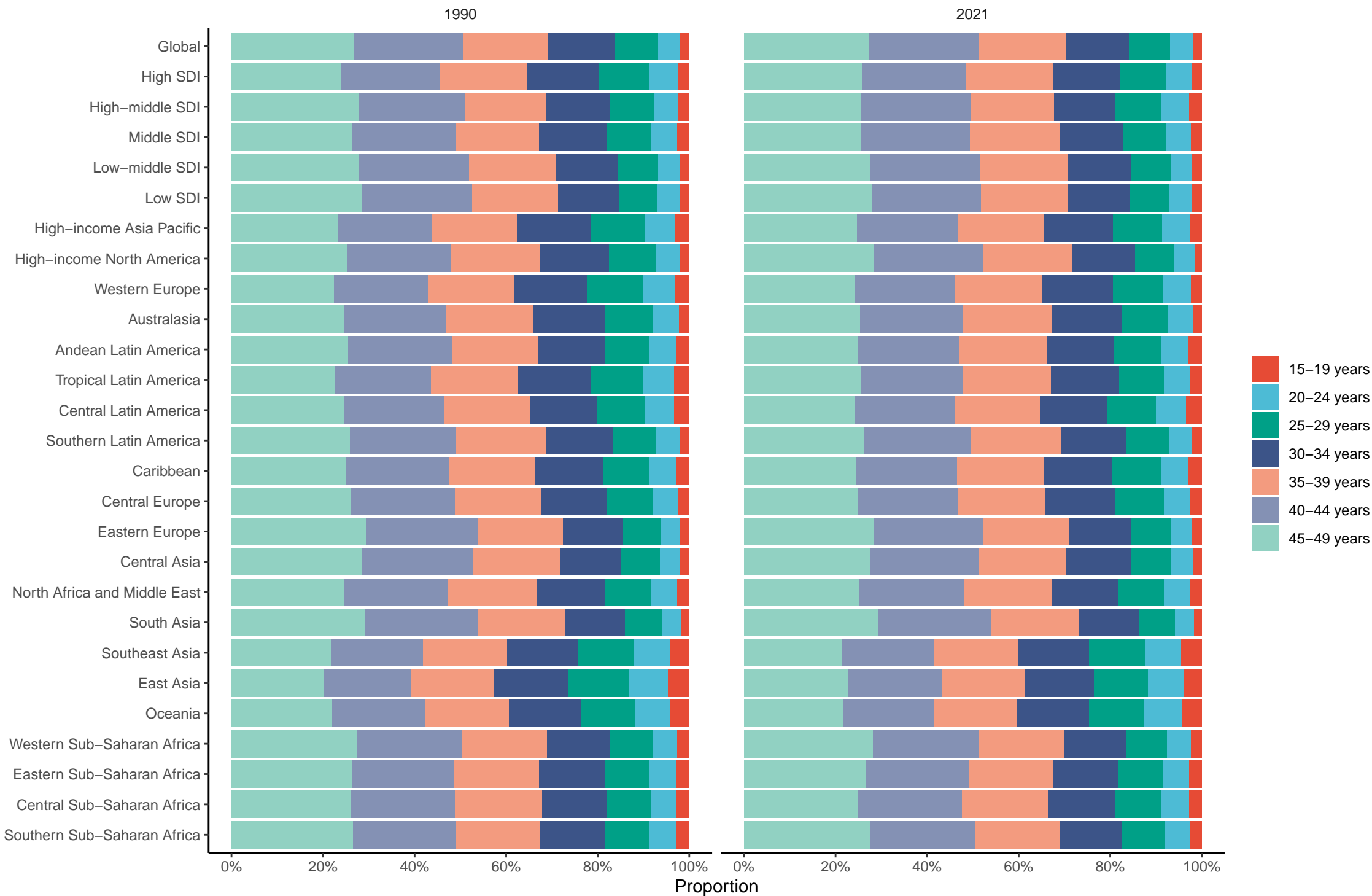

Supplement: Supplementary file 2 — Data S1: Supporting Information. [file JGH3-9-e70282-s002.zip › supplement material/Proportions of Age-Standardized Rates by Age Group/prevalence/Different Regions Proportions of Age-Standardized Prevalence Rates by Age Group.pdf]

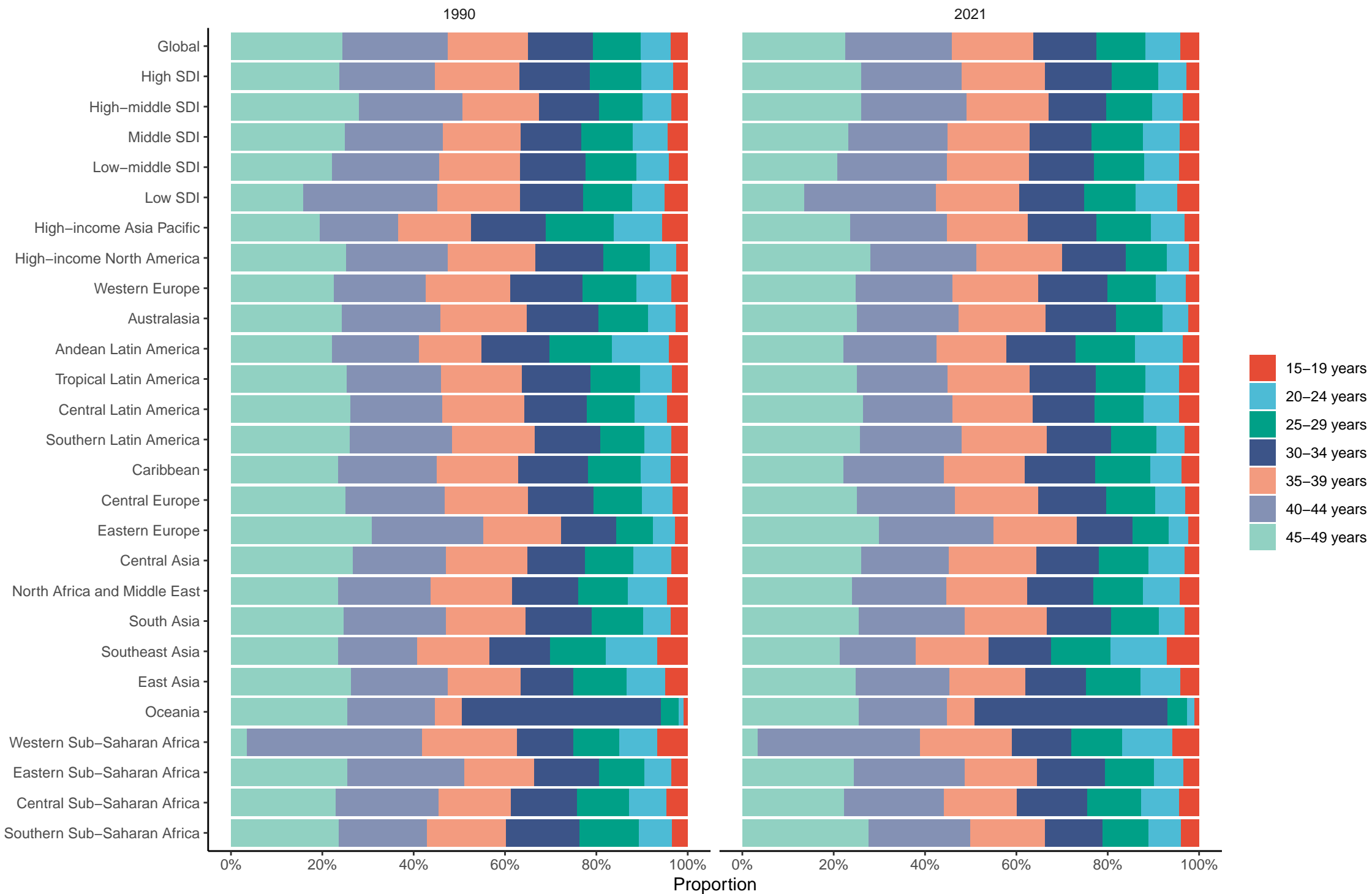

Supplement: Supplementary file 2 — Data S1: Supporting Information. [file JGH3-9-e70282-s002.zip › supplement material/Proportions of Age-Standardized Rates by Age Group/DALYs/Different Regions Proportions of Age-Standardized DALYs Rates by Age Group.pdf]

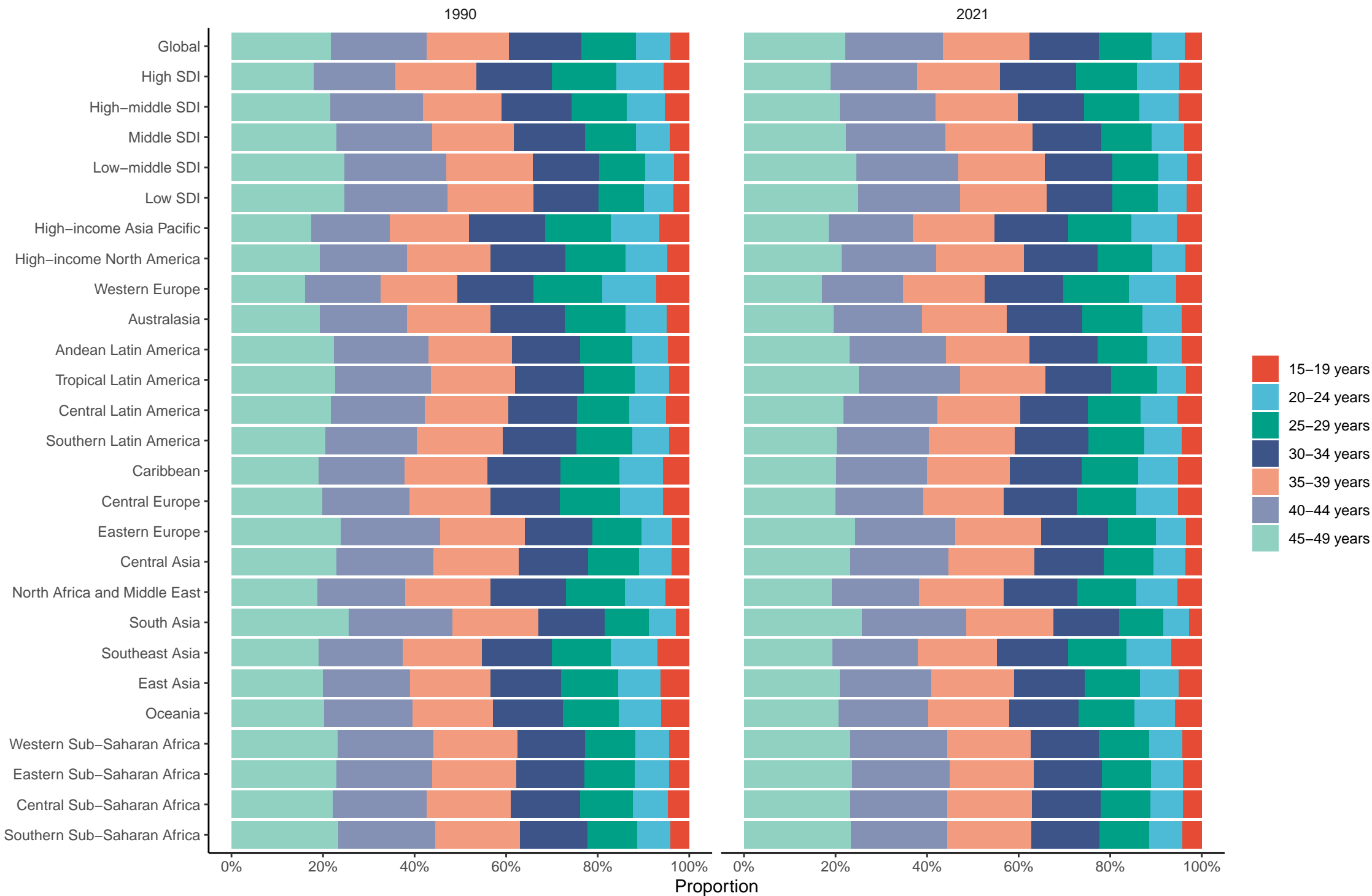

Supplement: Supplementary file 2 — Data S1: Supporting Information. [file JGH3-9-e70282-s002.zip › supplement material/Proportions of Age-Standardized Rates by Age Group/incidence/Different Regions Proportions of Age-Standardized Incidence Rates by Age Group.pdf]

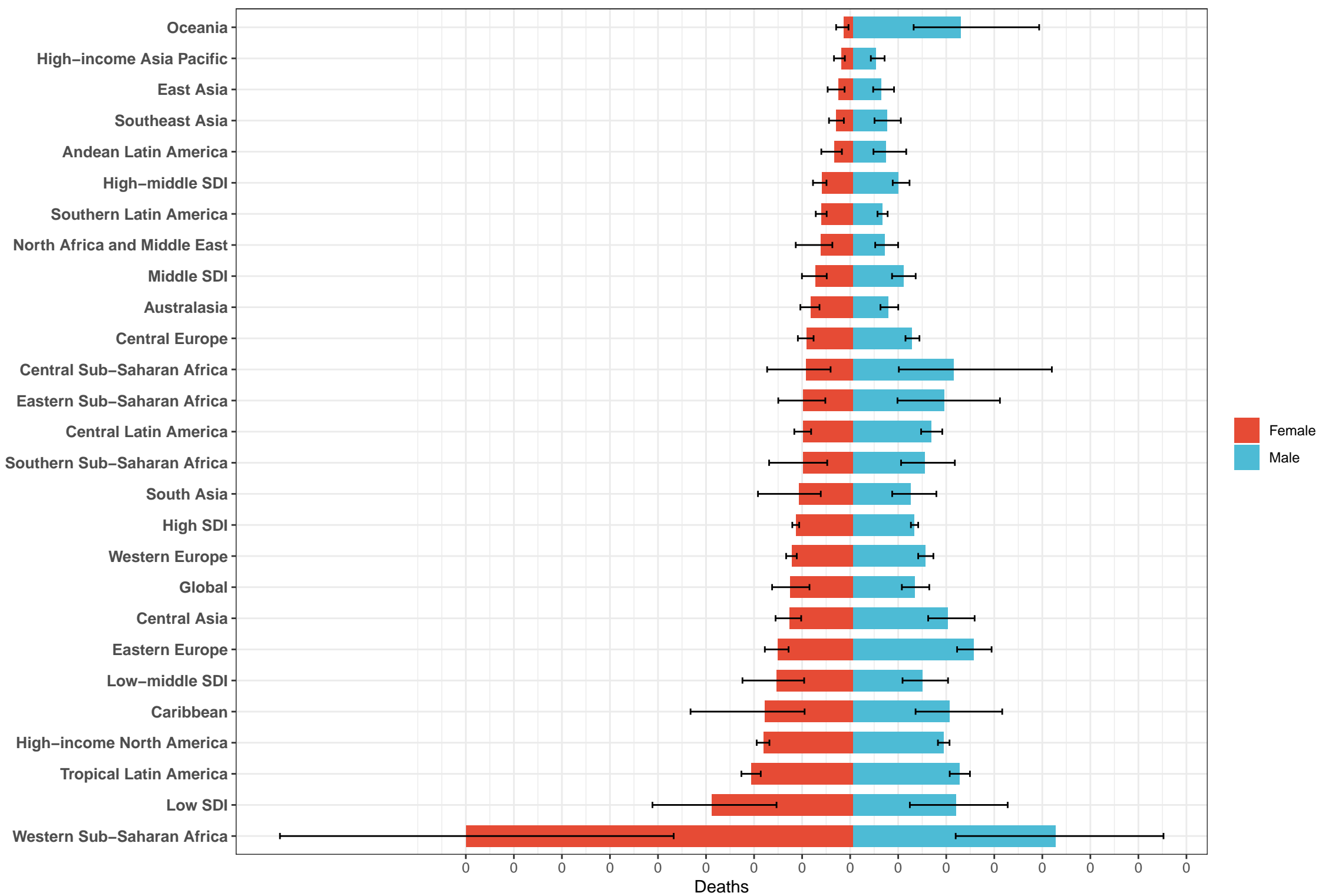

Supplement: Supplementary file 2 — Data S1: Supporting Information. [file JGH3-9-e70282-s002.zip › supplement material/Proportions of Age-Standardized Rates by Sex Group/death/Different Regions Proportions of Age-Standardized Mortality Rates by Sex Group.pdf]

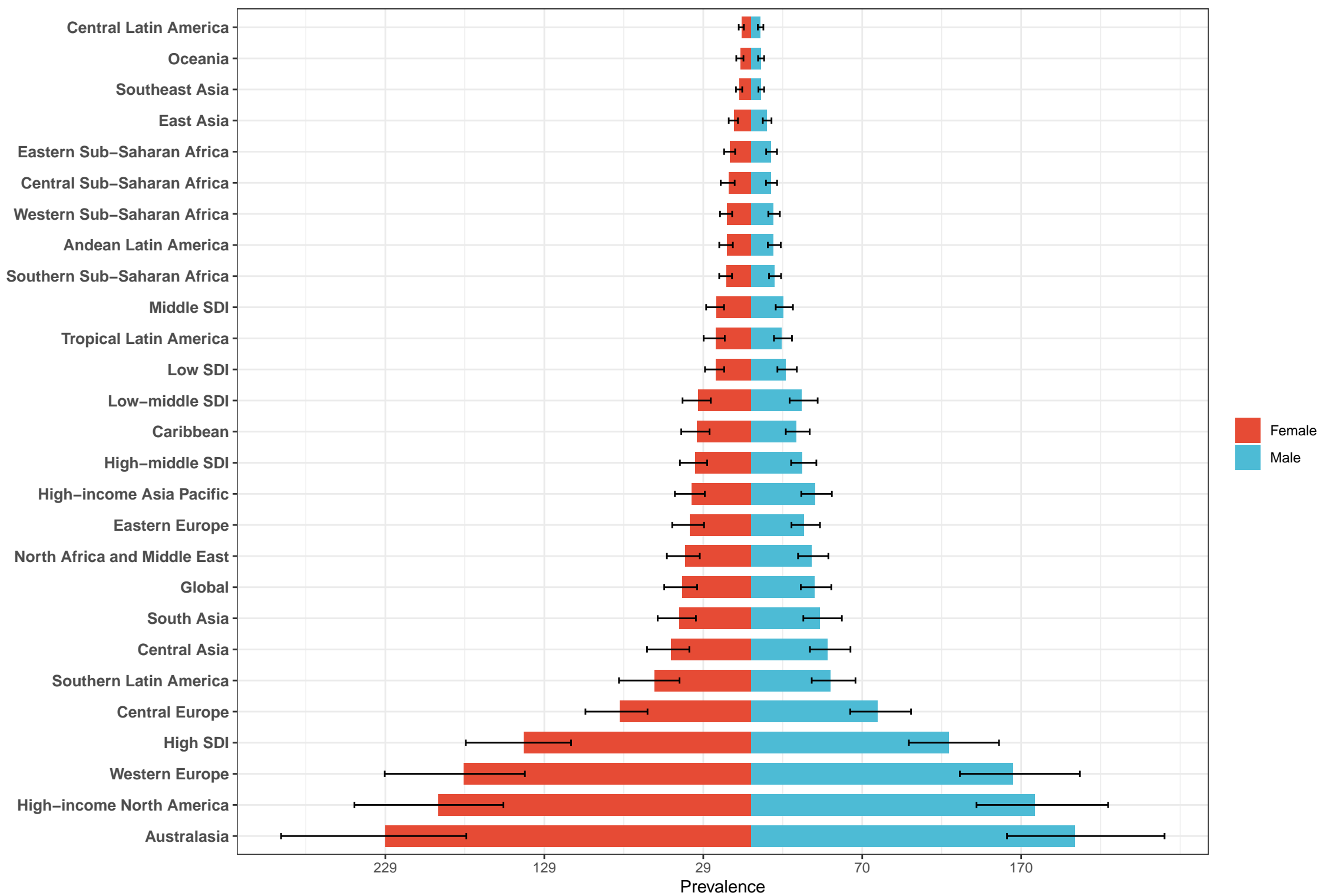

Supplement: Supplementary file 2 — Data S1: Supporting Information. [file JGH3-9-e70282-s002.zip › supplement material/Proportions of Age-Standardized Rates by Sex Group/prevalence/Different Regions Proportions of Age-Standardized Prevalence Rates by Sex Group.pdf]

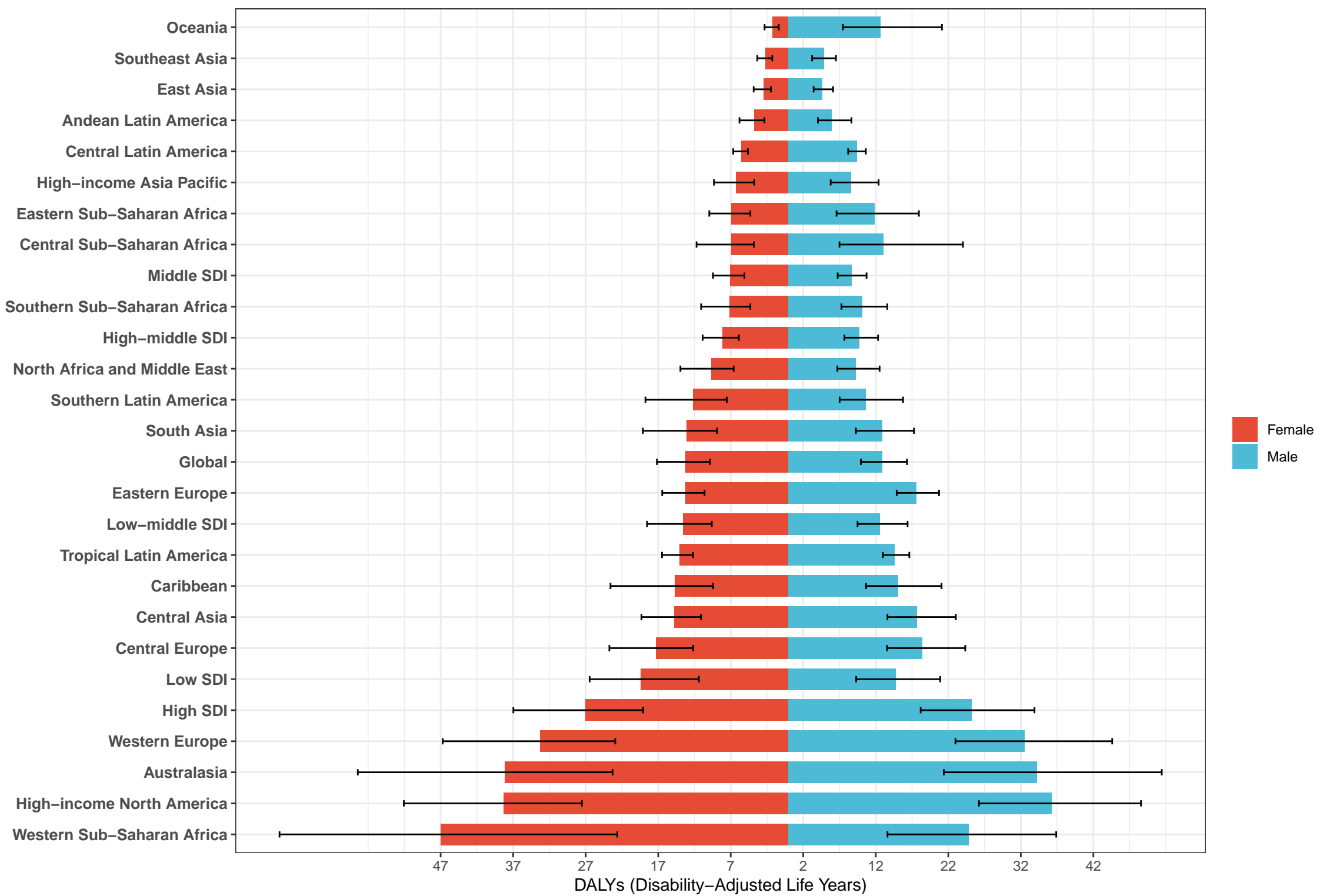

Supplement: Supplementary file 2 — Data S1: Supporting Information. [file JGH3-9-e70282-s002.zip › supplement material/Proportions of Age-Standardized Rates by Sex Group/DALYs/Different Regions Proportions of Age-Standardized DALYs Rates by Sex Group.pdf]

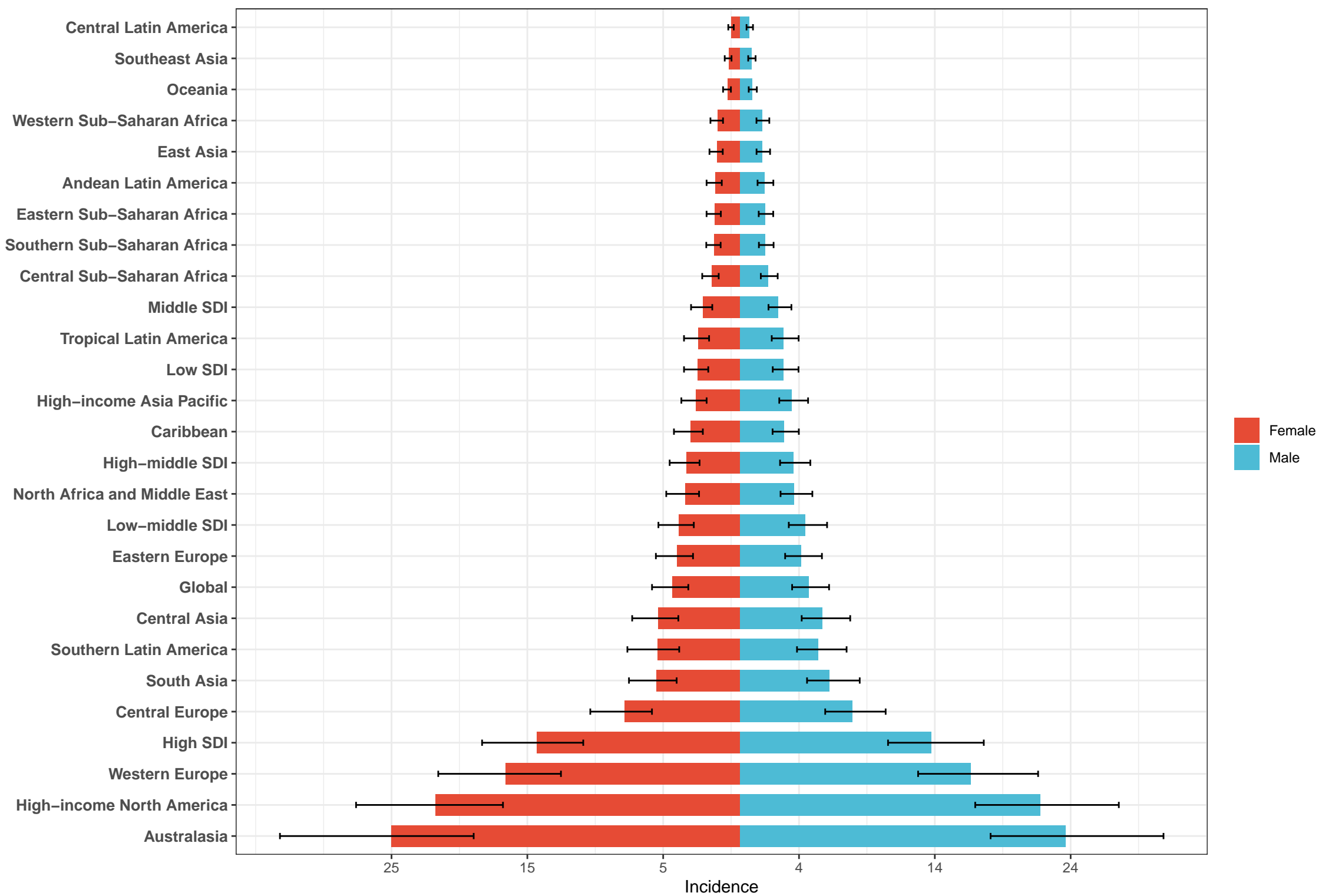

Supplement: Supplementary file 2 — Data S1: Supporting Information. [file JGH3-9-e70282-s002.zip › supplement material/Proportions of Age-Standardized Rates by Sex Group/incidence/Different Regions Proportions of Age-Standardized Incidence Rates by Sex Group.pdf]
